# The broad scale impact of climate change on planning aerial wildlife surveys with drone-based thermal cameras

**DOI:** 10.1038/s41598-023-31150-5

**Published:** 2023-03-17

**Authors:** Annalysa M. Camacho, Humberto L. Perotto-Baldivieso, Evan P. Tanner, Amanda L. Montemayor, Walter A. Gless, Jesse Exum, Thomas J. Yamashita, Aaron M. Foley, Randy W. DeYoung, Shad D. Nelson

**Affiliations:** 1 Caesar Kleberg Wildlife Research Institute, Texas A&M University-Kingsville, Kingsville, TX 78363 USA; 2 Dick and Mary Lewis Kleberg College of Agriculture and Natural Resources, Texas A&M University-Kingsville, Kingsville, TX 78363 USA

**Keywords:** Environmental sciences, Biogeography, Climate-change ecology

## Abstract

Helicopters used for aerial wildlife surveys are expensive, dangerous and time consuming. Drones and thermal infrared cameras can detect wildlife, though the ability to detect individuals is dependent on weather conditions. While we have a good understanding of local weather conditions, we do not have a broad-scale assessment of ambient temperature to plan drone wildlife surveys. Climate change will affect our ability to conduct thermal surveys in the future. Our objective was to determine optimal annual and daily time periods to conduct surveys. We present a case study in Texas, (United States of America [USA]) where we acquired and compared average monthly temperature data from 1990 to 2019, hourly temperature data from 2010 to 2019 and projected monthly temperature data from 2021 to 2040 to identify areas where surveys would detect a commonly studied ungulate (white-tailed deer [*Odocoileus virginianus*]) during sunny or cloudy conditions. Mean temperatures increased when comparing the 1990–2019 to 2010–2019 periods. Mean temperatures above the maximum ambient temperature in which white-tailed deer can be detected increased in 72, 10, 10, and 24 of the 254 Texas counties in June, July, August, and September, respectively. Future climate projections indicate that temperatures above the maximum ambient temperature in which white-tailed deer can be detected will increase in 32, 12, 15, and 47 counties in June, July, August, and September, respectively when comparing 2010–2019 with 2021–2040. This analysis can assist planning, and scheduling thermal drone wildlife surveys across the year and combined with daily data can be efficient to plan drone flights.

## Introduction

Traditionally, piloted aircrafts, such as helicopters, have been used to conduct aerial wildlife surveys^1–3^. However, these are expensive, dangerous, and time consuming^4–6^. The use of remotely piloted aerial vehicles (hereafter ‘drones’) has become a common method of data collection by ecologists, conservationists, and wildlife managers^5,7–10^. The use of drones in aerial wildlife surveys is one of the fastest growing segments in wildlife management and natural resources management. Drones are better suited for collecting data at fine spatial and temporal scales compared to conventional aircraft and spacecraft^7,8,11^. Drones can be used with autonomous computer vision algorithms to create high-resolution imagery and videography suitable for detecting and identifying a variety of flora and fauna^12,13^. Wildlife biologists are using drones to observe wildlife in environments ranging from tropical to polar climates, while quantifying the distribution and relative density of wildlife species^10,14,15^. Over the last decade, drones and their sensors have become more affordable, user-friendly, and can be flown at low altitudes. Finally, drones present the opportunity to survey areas quickly and repeatedly, a distinct advantage over other methods of aerial surveys^5,9,16–18^.

In recent years, thermal infrared sensors have improved in resolution, cost, and use in drone platforms, providing new opportunities for wildlife research^4,18^. Thermal infrared sensors rely on high contrast of body temperature to discriminate individuals from their surroundings and, unlike standard red, green, and blue (RGB) sensors, can be used at night^4,5,17^. Factors that influence the detection ability of thermal sensors are the distance from the animal, species size, vegetation cover between the target animal and the sensor, sensor properties, weather conditions, and time of year^5,17^. Drones and thermal infrared sensors have been used to detect ungulates^4,18^, avian nests^19^, marsupials^17,20^, primates^21^, and pinnipeds^22^. These studies highlight the importance of selecting the proper season and time of day to maximize thermal contrast when using thermal sensors in drones for detection and identification of wildlife species. The higher the thermal contrast, the better the opportunity to detect and potentially identify a species. If the contrast decreases or temperatures increase, it becomes more difficult to distinguish species as potential distortions can occur with image acquisition. These distortions can potentially affect annual counts or automation processes to estimate the number of observed individuals, which will propagate errors on population estimates derived from drones with thermal infrared sensors.

Drones have environmental requirements for flights (wind speed, operational ambient temperature) and the capabilities of thermal infrared sensors add to the restrictions of flight conditions for aerial wildlife surveys^23,24^. For example, wind speeds in coastal areas can be a flight safety limiting factor. Early daylight flights can be limited by weather conditions that can cause increased moisture or fog that can limit flight operations. Because thermal infrared sensors rely on high temperature contrasts between the target and its background^4,5^, it is important to conduct flights when these contrasts are optimal. Identifying appropriate seasons and time periods across the year can provide broad planning schedules and determine the feasibility of conducting flights based on species biological information. These schedules can then be refined with daily and hourly weather data to optimize drone operations and data acquisition.

When making projections using historical weather and species data, we must consider changes in climate patterns and potential impacts on surveys and data collection. Within the last 200 years, mean land surface air temperature has increased by 1.53 °C^25^. Changes in thermal variability are also evident, with increases in length, intensity, and frequency of heatwaves, increased intensity of heavy precipitation events, and increased intensity and frequency of droughts. Additionally, nighttime temperatures have increased more than daytime temperatures^26^, which can affect how ecological studies, particularly wildlife aerials surveys, may be conducted in the future. Due to increases in global temperatures, climate zones have shifted, expanding arid zones, and potentially affecting the distribution of many plant and animal species^25^. Therefore, modeling potential temperature changes based on climate change predictions can be useful to assess the logistical future of drone aerial surveys for the management and conservation of wildlife.

Considering the current and potential future constraints that accompany drone aerial surveys, development of a planning method for drone aerial wildlife surveys using thermal data is a critical need for the technology to be useful for wildlife and conservation research. Our goal was to develop a method to plan aerial surveys using drones and thermal cameras to detect wildlife based on ambient temperatures at different spatial and temporal scales. Specific objectives were (1) to develop a broad-scale analysis to plan for wildlife thermal surveys, and (2) to quantify the potential impact of climate change in the application of this broad-scale analysis. We present a case study for Texas, United States of America (USA), with a common and widespread ungulate (white-tailed deer [*Odocoileus virginianus*]; hereafter: “deer”) as our model species. We selected Texas because of the wide range in the annual and spatial temperature gradient and deer because of their economic and recreational importance and their large geographic distribution in the Western Hemisphere^27–29^. The approaches we present here can be used to develop similar analyses across taxonomic groups and disparate biomes.

## Methods

### Study area

**Figure 1:**
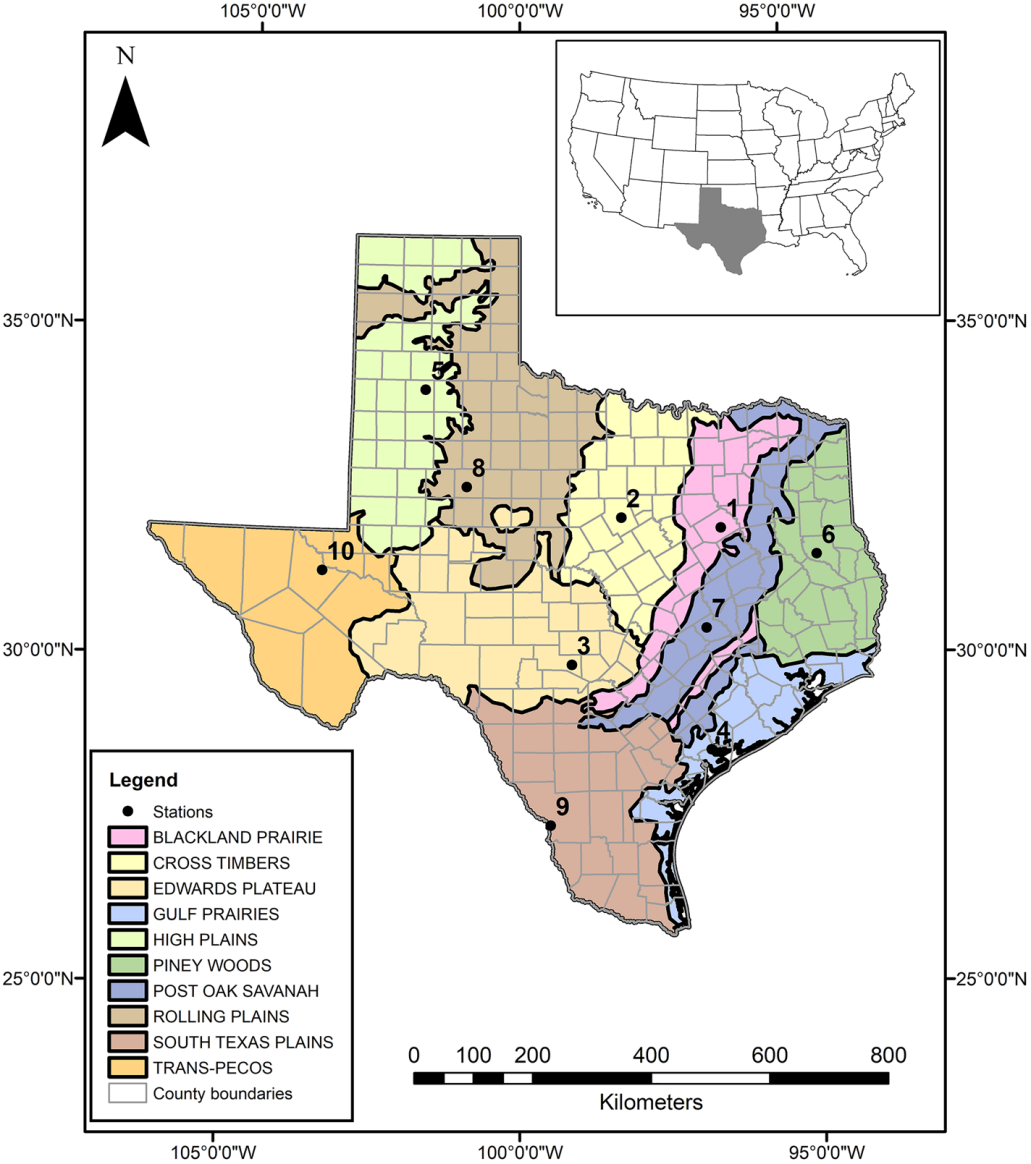
Map showing Texas Ecoregions^30^ (Texas Parks and Wildlife Department) and selected counties (www.tnris.org) for hourly data analysis. The points in the map represent the location of each station. The numbers refer to the station names: 1: Corsicana Campbell Field Municipal Airport; 2: Stephenville Clark Regional Airport; 3: Kerrville-Kerr County Airport; 4: Calhoun County Airport; 5: Plainview Hale County Airport; 6: A. L. Mangham Jr. Regional Airport; 7: Caldwell Municipal Airport; 8: Winston Field Airport; 9: Laredo International Airport; 10: Pecos Municipal Airport. The inset provides the location of the State of Texas within the Continental United States of America (U.S. Census Bureau). The map was generated in ArcMap 10.8.1 (www.esri.com).

Texas has 254 counties and encompasses 695,621 km^2^^30^. There are 10 level III ecoregions: the Pineywoods, Gulf Prairies and Marshes, Post Oak Savannah, Blackland Prairies, Cross Timbers and Prairies, South Texas Plains, Edwards Plateau, Rolling Plains, High Plains, and the Trans-Pecos Mountains and Basins (Fig. 1). There is a general climatic trend across the State; from east-to-west, annual mean precipitation decreases, and from north-to-south, annual mean temperature increases^31,33^. Typical daily minimum temperatures in January range from − 6 °C in the north to 10 °C in the southern parts of the state. Typical daily maximum temperatures in July are more consistent from north to south (33–35 °C), with an adiabatic lapse rate in the mountains of West Texas (28–31 °C).

### Climate data collection

We acquired 30 years (1990–2019) of average monthly temperature data for each county in Texas from the National Oceanic and Atmospheric Administration’s (NOAA) U.S. Climate Divisional Database through the Climate at a Glance, County Time Series tool (https://www.ncdc.noaa.gov/cag/county/time-series). We subsampled the 30-year dataset to analyze a 10-year dataset (2010–2019) to determine if there were increased mean monthly temperatures in the last 10 years compared to the last 30 years. We also downloaded 10 years (2010–2019) of average hourly temperature data from NOAA’s Local Climatological Data summaries (https://www.ncdc.noaa.gov/cdo-web/datatools/lcd). We selected one station per ecoregion based on data availability of complete hourly datasets (Fig. 1; Table 1): Burleson, Calhoun, Erath, Hale, Kerr, Nacogdoches, Navarro, Reeves, Scurry, and Webb counties.

**Table 1:** Weather stations used to collect hourly data.

Station number	Station name	City, county	Ecological region	Coordinates
1	Corsicana Campbell Field Municipal Airport	Corsicana, Navarro Co	Blackland Prairie	32.03111°, − 96.39889°
2	Stephenville Clark Regional Airport	Stephenville, Erath Co	Cross Timbers	32.21528°, − 98.1775°
3	Kerrville-Kerr County Airport	Kerrville, Kerr Co	Edwards Plateau	29.98333°, − 99.08333°
4	Calhoun County Airport	Port lavaca, Calhoun Co	Gulf Prairies	28.65417°, − 96.68139°
5	Plainview Hale County Airport	Plainview, Hale Co	High Plains	34.16667°, − 101.71667°
6	A. L. Mangham Jr. Regional Airport	Nacogdoches, Nacogdoches Co	Piney Woods	31.57778°, − 94.70944°
7	Caldwell Municipal Airport	Caldwell, Burleson Co	Post Oak Savanah	30.51556°, − 96.70417°
8	Winston Field Airport	Snyder, Scurry Co	Rolling Plains	32.69333°, − 100.95056°
9	Laredo International Airport	Laredo, Webb Co	South Texas Plains	27.53333°, − 99.46667°
10	Pecos Municipal Airport	Pecos, Reeves Co	Trans-Pecos	31.3825°, − 103.51083°

To represent future climatic conditions, we acquired Coupled Model Intercomparison Project Phase 6 (CMIP6) files from the WorldClim database (https://www.worldclim.org/), at a spatial resolution of 2.5 min. General circulation models (GCMs) use emission scenarios of greenhouse gasses to forecast future weather and climate change. The WorldClim database uses downscaled climate data, which predicts the change in a weather variable (i.e. monthly minimum and maximum temperatures) as the difference between the output of the global climate models for the baseline years (usually 1960–1990 for future climate studies) and for the target years (2021–2040) (www.worldclim.org). These changes are interpolated to a high-resolution (1 km) grid, then calibrated to high-resolution interpolated current climate data (WorldClim v2.1). The WorldClim database provides monthly minimum temperature (°C), maximum temperature (°C), and precipitation (mm) for nine GCMs and four shared socio-economic pathways (SSPs) for 2021–2100 (in 20-year time intervals). These SSPs are based on socioeconomic trends that are plausible in the future: SSP1 predicts a world practicing sustainable growth and social equality; SSP2 predicts little to no change in practices; SSP3 predicts fast-growing populations and increased inequalities with high challenges to mitigation and adaptation; and SSP5 predicts a world focused on fossil-fueled development with high energy consumption^33^.

We used three GCMs projections, The Canadian Earth System Model version 5 (Can-ESM5)^34^ (Figs. S1–S4), the Institut Pierre-Simon Laplace- Climate Model version 6A- Low Resolution (IPSL-CM6A-LR)^35^ (Figs. S5–S8), and the Model for Interdisciplinary Research on Climate, Earth System version 2 for Long-term simulations (MIROC-ES2L)^36^ (Figs. S9–SS12). We selected these three GCMs because they ranged from high (Can-ESM5) to low (MIROC-ES2L) “climate sensitivity”^37^ based on the models available. Climate sensitivity refers to the changes in global surface temperatures based on CO_2_ emissions compared to pre-industrial levels (estimated to be 1.5–4.5 °C). For each GCM and SSP, we obtained minimum and maximum temperature for the 2021–2040 time period, resulting in a total of 12 GCM and SSP combinations.

### Data analysis

**Figure 2:**
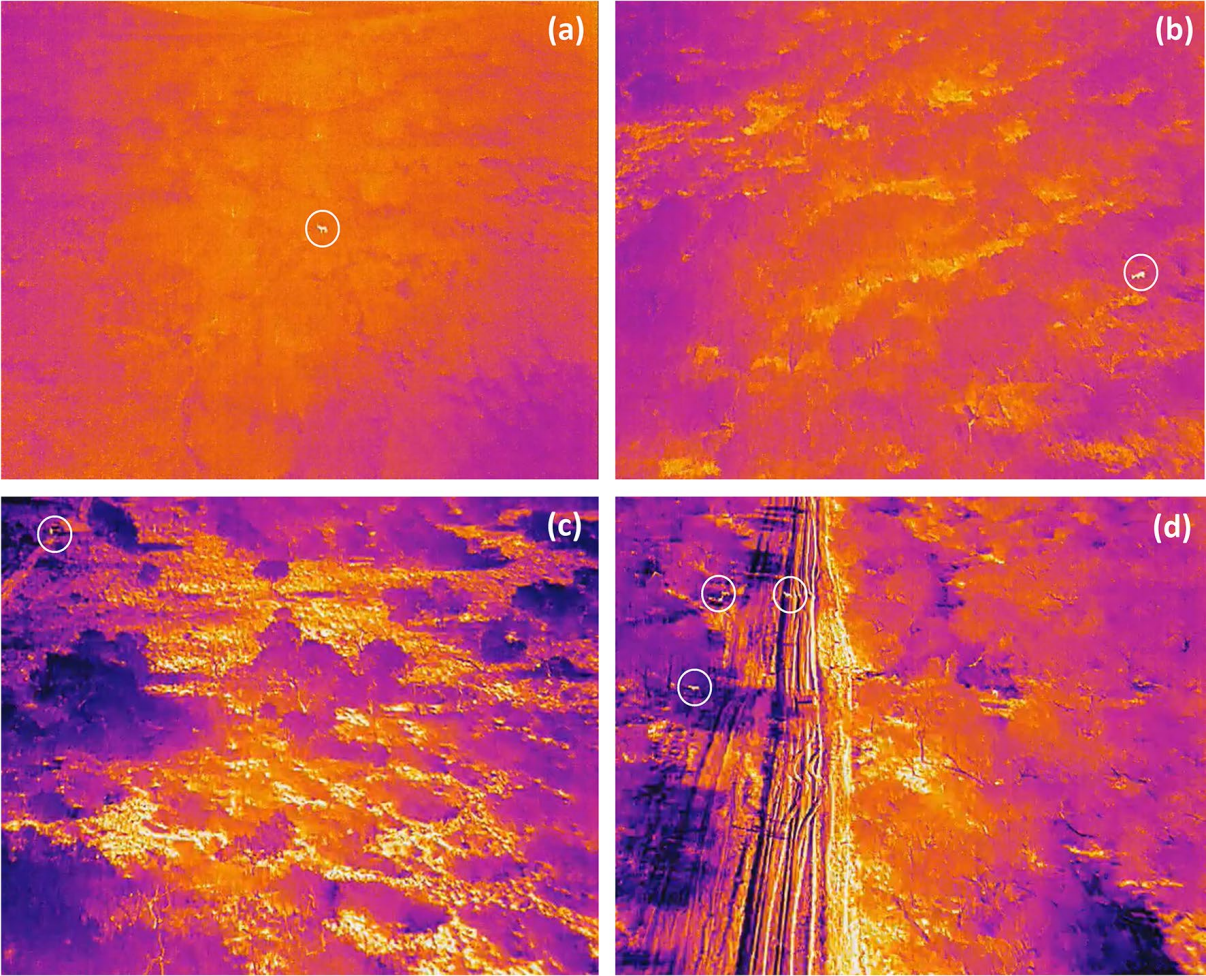
Thermal images indicating white-tailed deer (*Odocoileus virginianus*) detections from video footage during drone surveys in South Texas, USA in 2020. The top images highlight detections below (**a**) 20 °C and (**b**) 27 °C on a sunny and cloudy days respectively. The bottom images highlight detections above (**c**) 20 °C and (**d**) 27 °C on a sunny and cloudy days respectively. White circles indicate white-tailed deer detections. Orange and yellow areas were hotter and deer within these areas would not be detected.

There is a limited number of peer-reviewed publications that addresses the maximum ambient temperature in which deer can be detected using drones over a broad geographic region. Preston et al.^38^ conducted drone aerial deer surveys in forested areas of Virginia with ambient temperatures ranging from 8 to 13 °C, and observed that deer were only detectable on cloudy days. We established the maximum temperatures (20 °C for clear days and 27 °C for cloudy days) at which deer can be distinguished throughout the entire viewshed using a commercially available drone thermal camera (DJI FLIR Zenmuse XT2 Thermal Camera) during early daylight hours based on empirical observations for South Texas^39^ (Fig. 2) and previous literature on thermal detections for other mammals^40,41^. Ambient temperatures above 20 °C on clear days (hereafter ‘maximum clear ambient temperature’) and 27 °C on cloudy days (hereafter ‘maximum cloudy ambient temperature’) decreased the ability to visually identify individuals because of distortions in the detection of the species.

For the monthly data analysis, we reclassified monthly temperature data for each county using observed maximum ambient temperatures for detecting deer: ≤ 20 °C for clear days and ≤ 27 °C for cloudy days. We classified counties as detectable (≤ 20 °C), detectable when cloudy (20–27 °C), and non-detectable (> 27 °C). We compared the 1990–2019 and 2010–2019 monthly data to determine if there were temperature increases in the last 10 years compared to the 30-year mean. For the hourly data analysis, we quantified the mean ambient temperature for hourly temperatures aggregated by month.

For the future climate projections, we summarized the minimum and maximum monthly temperature from each GCM and SSP combination for each county in Texas using zonal statistics in ArcMap 10.8 (ESRI, The Redlands). We averaged these minimum and maximum temperatures to obtain the average monthly temperature. We then created an ensemble model by taking the average of all the GCM and SSP combinations^42^ (Fig. S13). Monthly data from 1990 to 2019, 2010–2019, and future climate projections from 2021 to 2040 were aggregated at the county level to compare mean temperatures to develop temperature maps for Texas for each time period. For future hourly forecasts, we used future microclimate models developed by Levy et al.^43^ to predict the mean hourly temperatures for each month at each location (Table 1) averaged from 2080 to 2100. We dynamically downscaled a previously published, bias-corrected prediction of a global-circulation model (Community Earth System Model [CESM1]) to obtain a finer spatial (36 × 36 km) and temporal (hourly data) dataset than previous models (100 × 130 km). Estimated mean hourly differences per month were added to our current (2010–2019) hourly means to indicate the potential changes in temperature projected to the 2080–2100 period. Estimates of future air temperature values were calculated assuming 0% vegetation shade and a measurement height of 1.53 m above ground level, which is the typical measurement height used to measure air temperature at NOAA weather stations. To our knowledge, this is the only dataset and model that can provide hourly forecasted weather data within North America to meet the scope of our research.

## Results

**Figure 3:**
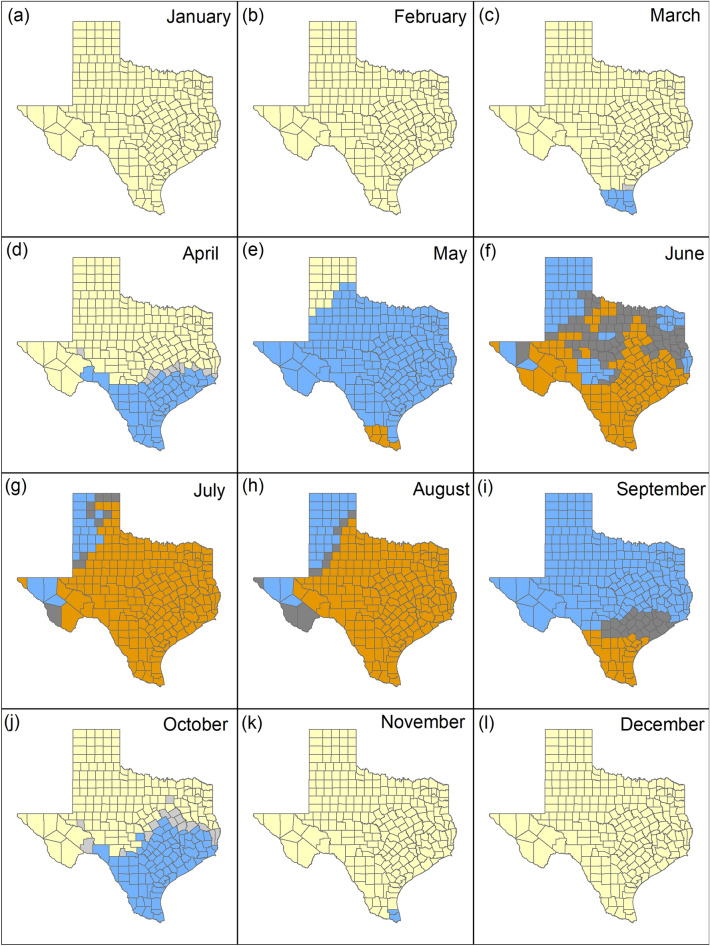
Monthly mean temperature (°C) from 1990 to 2019 compared to 2010–2019 for Texas aggregated by county. Yellow counties represent areas where temperatures are below the maximum clear ambient temperature in which deer can be detected (≤ 20 °C), blue counties represent areas where temperatures are below the maximum cloudy ambient temperature in which deer can be detected (≤ 27 °C), and orange counties represent areas where temperatures are above the maximum ambient temperatures for deer detection (> 27 °C). Light gray counties represent areas where temperatures increased from clear to cloudy maximum ambient temperatures and dark gray counties represent areas where temperatures increased above 27 °C.

Overall mean temperature in Texas increased from 18.7 °C (SE = 0.14 °C) for the period 1990–2019 (30-year) to 19.0 °C (SE = 0.13 °C) for the period 2010–2019 (10-year). Mean monthly temperatures from 30-year and 10-year data show that in January (Fig. 3a), February (Fig. 3b), and December (Fig. 3l), the temperatures are below the maximum clear ambient temperature in which deer can be detected in all counties in Texas. During the months of March (Fig. 3c), April (Fig. 3d), October (Fig. 3j) and November (Fig. 3k) temperatures were below the maximum clear ambient temperature (< 20 °C) or maximum cloudy ambient temperature (20° to 27 °C) for the 30- and 10-year data in all counties in Texas. In May, temperatures were below the maximum clear and cloudy ambient temperature in which deer can be detected across the state except for 6/254 (2.4%) southern counties (Brooks, Cameron, Hidalgo, Jim Hogg, Starr, Zapata) for the 30-year and 10-year data (Fig. 3e).

**Figure 4:**
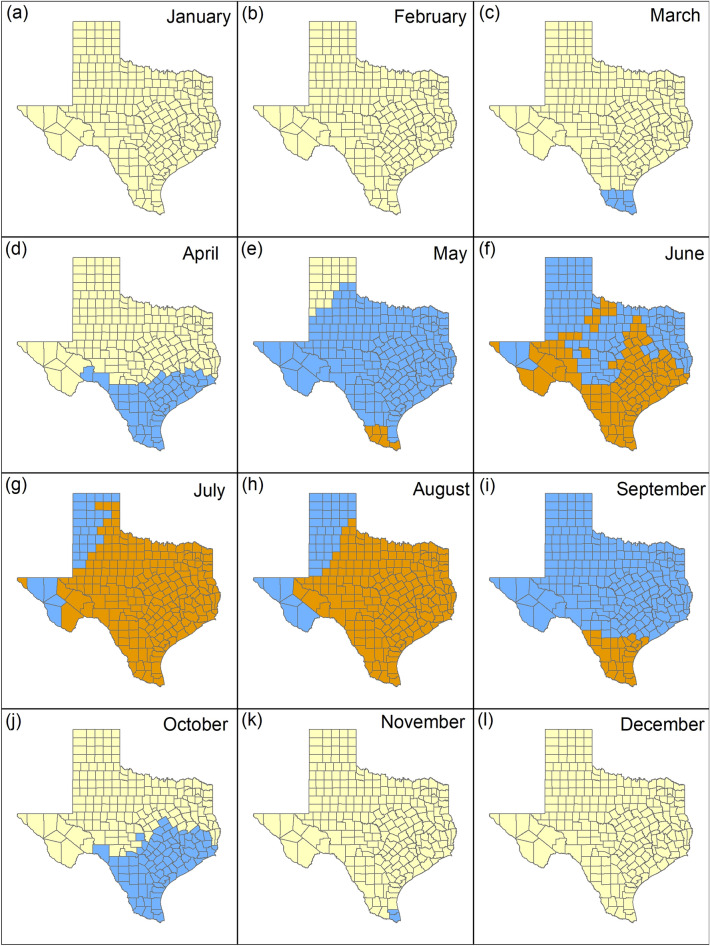
Monthly mean temperature (°C) from 1990 to 2019 for Texas aggregated by county. Yellow counties represent areas where temperatures are below the maximum clear ambient temperature in which deer can be detected (≤ 20 °C), blue counties represent areas where temperatures are below the maximum cloudy ambient temperature in which deer can be detected (≤ 27 °C), and orange counties represent areas where temperatures are above the maximum ambient temperatures for deer detection (> 27 °C).

With the monthly 30-year data, temperatures were below the maximum clear or cloudy ambient temperatures in all counties between January and April (Fig. 4a–d) and October to December (Fig. 4j–l) and in 248/254 (97.3%) counties in May (Fig. 4e). Temperatures below the maximum cloudy ambient temperature in which deer can be detected were present in 136/254 (53.5%) counties in June (Fig. 4f), 31/254 (12.2%) counties in July (Fig. 4g), 43/254 (16.9%) counties in August (Fig. 4h), and 229/254 (90.1%) counties in September (Fig. 4i). With the monthly 10-year data, temperatures below the maximum cloudy ambient temperature in which deer can be detected were present in 64/254 (25.2%) counties in June (Fig. S14f.), 21/254 (8.3%) counties in July (Fig. S14g), 33/254 (13%) counties in August (Fig. S14h), and 205/254 (80.7%) counties in September (Fig. S14i). When comparing the 30-year to the 10-year data, temperatures would increase above the maximum ambient temperature in which deer can be detected in an additional 72/254 (28.3% in June; Fig. 3f), 10/254 (4% in July; Fig. 3g), 10/254 (4% in August; Fig. 3h), and 24/254 counties (9.4% in September; Fig. 3i).

**Figure 5:**
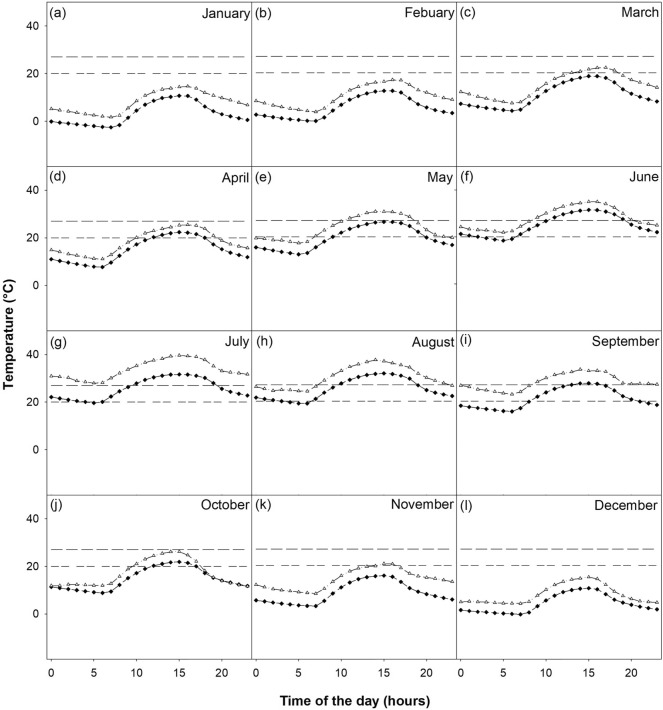
High Plains ecoregion hourly mean temperature by month between 2010 and 2019 (black diamonds) and projections to 2080 (white triangles). The double dashed line (20 °C) represents maximum clear ambient temperature and the single dashed line (27 °C) represents the maximum cloudy ambient temperature for deer detections.

**Figure 6:**
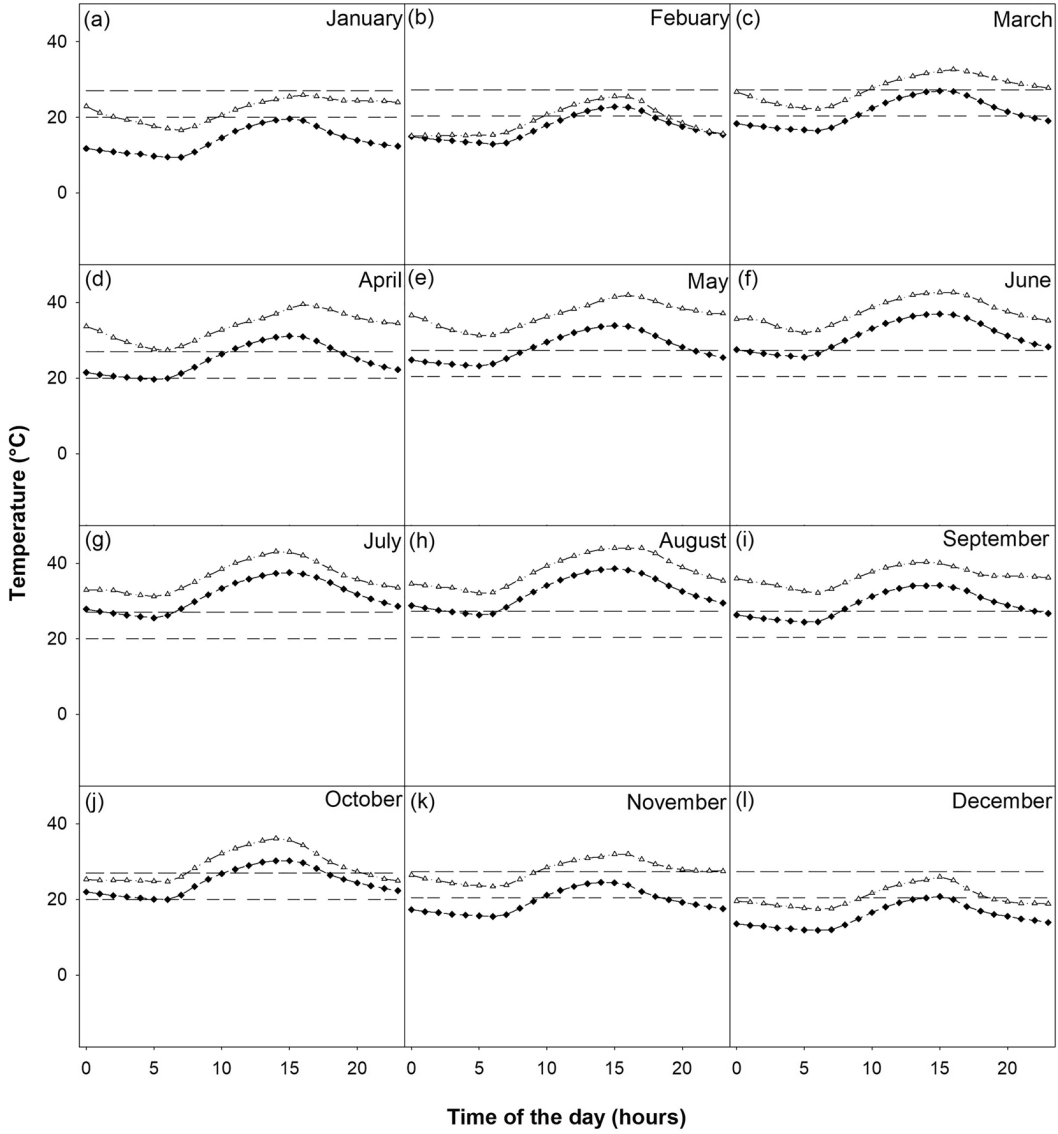
South Texas Plains ecoregion hourly mean temperature by month between 2010 and 2019 (black diamonds) and projections to 2080 (white triangles). The double dashed line (20 °C) represents maximum clear ambient temperature and the single dashed line (27 °C) represents the maximum cloudy ambient temperature for deer detections.

Hourly ambient temperature data indicate that favorable flight conditions decrease from northeast to southwest. For instance, in the High Plains ecoregion, temperatures were below the maximum cloudy ambient temperature at any hour from September to May and below the maximum clear ambient temperature from November to March (Fig. 5). In the Cross Timbers, Piney Woods, Blackland Prairies, and Rolling Plains ecoregions, temperatures below the maximum cloudy ambient temperature in which deer can be detected were present at any hour from October to May and below the maximum clear ambient temperature in which deer can be detected from November to March (Figs. S15, S16, S17, S18). In the Post Oak Savannah and Edwards Plateau ecoregions, temperatures below the maximum cloudy ambient temperature in which deer can be detected were present at any hour from October to April (Figs. S19, S20). In the Post Oak Savannah, temperatures below the maximum clear ambient temperature in which deer can be detected were present at any hour under November to March (Fig. S19). In the Edwards Plateau ecoregion, temperatures below the maximum ambient temperature in which deer can be detected were present from November to February (Fig. S20). In the Trans-Pecos ecoregion, maximum cloudy ambient temperatures in which deer can be detected were present at any hour from October to March and temperatures below the maximum clear ambient temperature in which deer can be detected, from November to February (Fig. S21). In the Gulf Prairies ecoregion, maximum cloudy ambient temperatures in which deer can be detected were present at any hour from November to April and temperatures below the maximum clear ambient temperature in which deer can be detected, from December to February (Fig. S22). In the South Texas Plains ecoregion, maximum ambient temperatures in which deer can be detected were present at any hour from November to March under cloudy conditions and in December and January under clear conditions (Fig. 6). Hourly ambient temperature data forecasted to the 2080–2100 period indicate that favorable flight conditions will decrease from northeast to southwest. Favorable conditions will be available from October to May in the High Plains ecoregion (Fig. 5), from October to April in the Piney Woods (Fig. S16), Edwards Plateau (Fig. S20), and Trans-Pecos (Fig. S21) ecoregions, and from November to February in the South Texas Plains ecoregion (Fig. 6).

**Figure 7:**
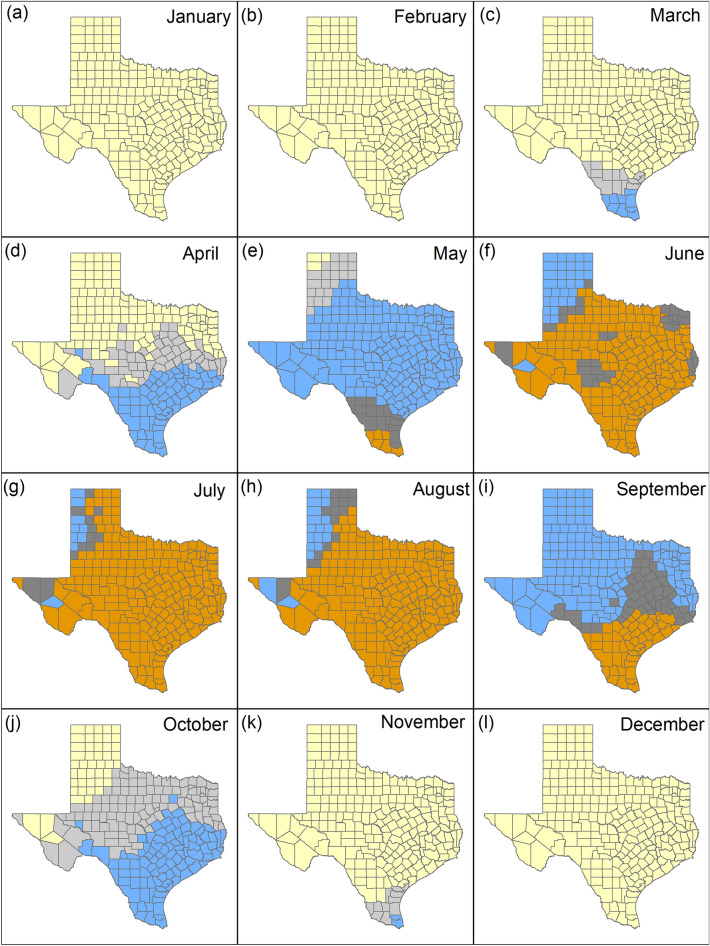
Monthly mean temperature (°C) from 2010 to 2019 compared to ensemble model 2021–2040 projections aggregated by county. Yellow counties represent areas where temperatures are below the maximum clear ambient temperature in which deer can be detected (≤ 20 °C), blue counties represent areas where temperatures are below the maximum cloudy ambient temperature in which deer can be detected (≤ 27 °C), and orange counties represent areas where temperatures are above the maximum ambient temperatures for deer detection (> 27 °C). Light gray counties represent areas where temperatures increased from clear to cloudy maximum ambient temperatures and dark gray counties represent areas where temperatures increased above 27 °C.

The CanESM5 SSP 5–8.5 model predicted the greatest temperature changes (Fig. S23) and the IPSL-CM6A-LR SSP 1–2.6 model predicted the lowest temperature changes (Fig. S24). When comparing our ensemble model (Fig. S25) to the 10-year data (Fig. S14), no changes were observed in January (Fig. 7a), February (Fig. 7b), and December (Fig. 7l). Temperatures would increase above the maximum cloudy ambient temperature in which deer can be detected in and additional 13 counties (5.1% of Texas counties) in March (Fig. 7c), 63 counties (24.8% of Texas counties) in April (Fig. 7d), 111 counties (43.7% of Texas counties) in October (Fig. 7j), and 11 counties (4.3% of Texas counties) in November (Fig. 7k). Temperatures would increase above the maximum ambient temperatures in which deer can be detected in an additional 22 (cloudy conditions) and 16 counties (clear conditions) (8.7% and 6.3% of Texas counties respectively) in May (Fig. 7e). Temperatures would increase above the maximum ambient temperatures in which deer can be detected in an additional 32 counties (12.6% of Texas counties) in June (Fig. 7f), 12 counties (4.7% of Texas counties) in July (Fig. 7g), 15 counties (6% of Texas counties) in August (Fig. 7h), and 47 counties (18.5% of Texas counties) in September (Fig. 7i).

## Discussion

Drones are a flexible and accessible tool for wildlife surveys, yet are limited by flight conditions; the additional requirements of thermal infrared sensors for detection pose further constraints on their use. Understanding annual temperature patterns at broad scales can improve the planning and organization of drone-based aerial surveys. Our research provides a broad-scale analysis for drone-based surveys using deer in Texas as a case study. We found the number of flight hours and conditions where deer would be detectable decreased from north to south and east to west. October through April may be the best time to conduct daylight flights under clear or cloudy conditions in most of Texas. There is little research that addresses temperature requirements for wildlife surveys^5^, and most of this research focuses on local scales. The development of operational protocols combined with broad-scale planning will pave new opportunities to develop objective approaches to wildlife aerial surveys using drones. The use of drones in deer surveys has been shown to provide accurate estimates^44^. These accurate estimates open new opportunities to estimate populations at different times of the year. Helicopter-based deer surveys in Texas are usually conducted during autumn or winter^2,45,46^ before or after the bulk of recreational harvest. Our findings indicate that thermal contrast would be too poor for drone surveys during autumns which suggest that crewed helicopters with would be the preferred platform. However, temperatures were below thermal thresholds during winter months which indicate that drones could be a viable platform given that drones have been found to generate accurate population estimates of deer^39,44^. While helicopter surveys are currently considered the most practical method to monitor deer, they are also expensive, dangerous, and time consuming^47,48^. However, the results of our study could be easily applied to helicopters and airplanes that use thermal sensors to study and quantify wildlife^49–51^. As technology develops, more sensitive instruments will allow for better contrasts as well as the use of automated processes to identify wildlife from still images and video^52^.

Global trends in climate change will affect our ability to detect wildlife species by reducing the extent and time period where detectability is optimal throughout the year. Southeast Texas temperatures are projected to rise steadily^53^ and northwest temperatures are projected to rise from 2.4° to 4.2 °C under different scenarios. Our analyses suggest that average temperatures in Texas over the last 10 years have increased by 0.3 °C, decreasing the number of counties where deer would be detectable for drone-based thermal wildlife surveys. Our projections suggest average temperatures will continue to increase, which will decrease the number of counties where deer would be detectable depending on the time of year. Changes in temperature will also affect wildlife and vegetation species distributions and may negatively affect species that are unable to adapt to these changes. Changes in climate patterns will have a wide impact on wildlife and vegetation and also on almost every aspect of natural resource management^54,55^.

Temperature changes will also have an impact on research and management operations. Devices used for surveys and research, such as smartphones, tablets and drones, have operating temperatures or temperature ranges at which the device can operate. When these devices are used outside of these technical specifications, devices can malfunction, become inaccurate, or shut off. For example, one of the most common drones (Matrice 210, DJI) used in aerial wildlife surveys, has an operating temperature of − 20 to 45 °C (https://www.dji.com/matrice-200-series/info). This can be a limiting factor during summer months in tropical and sub-tropical environments and reduce the ability to use this equipment unless technological advances provide a wider range of operating temperatures. The increase of extreme temperatures, and changes in wind and precipitation patterns may further exacerbate our ability to conduct aerial wildlife surveys in the future. Night flights may be an opportunity to expand planning operations throughout the year and reduce the impact of climate change on aerial wildlife surveys. However, specific nation and state regulations on the use of drones and airspace may be a limiting factor. As technology improves, drone flight time will increase, thermal sensor capabilities may improve, and drone-based aerial surveys may provide alternative approaches to current aerial wildlife methodologies. Similar to drones, operating temperature of other devices used in the field of natural resources will need to be adapted to their use in a changing environment.

Although we used white-tailed deer as our model species, this broad-scale analysis can be applied to different species in a variety of environments. Our results can be translated not only to other species, but to other technologies and other settings where extreme temperatures can be a limiting factor to conduct fieldwork. This broad-scale analysis aims at improving planning and scheduling effective aerial surveys for wildlife studies. This approach can be broadly applied to the field to understand where mismatches may exist between climatic change and technological limitations in conservation, agriculture, and technology development.

## Supplementary Information

Supplementary Figures.

## Data Availability

The datasets generated during and/or analyzed during the current study are available from the corresponding author on reasonable request.
