# Supplementary material for: The broad scale impact of climate change on planning aerial wildlife surveys with drone-based thermal cameras
Source: Sci Rep. 2023 Mar 17;13:4455. doi: 10.1038/s41598-023-31150-5 (PMC10023802; doi:10.1038/s41598-023-31150-5)
Supplement: Supplementary file 1 — Supplementary Figures. [file 41598_2023_31150_MOESM1_ESM.docx]

**Supplementary Material**


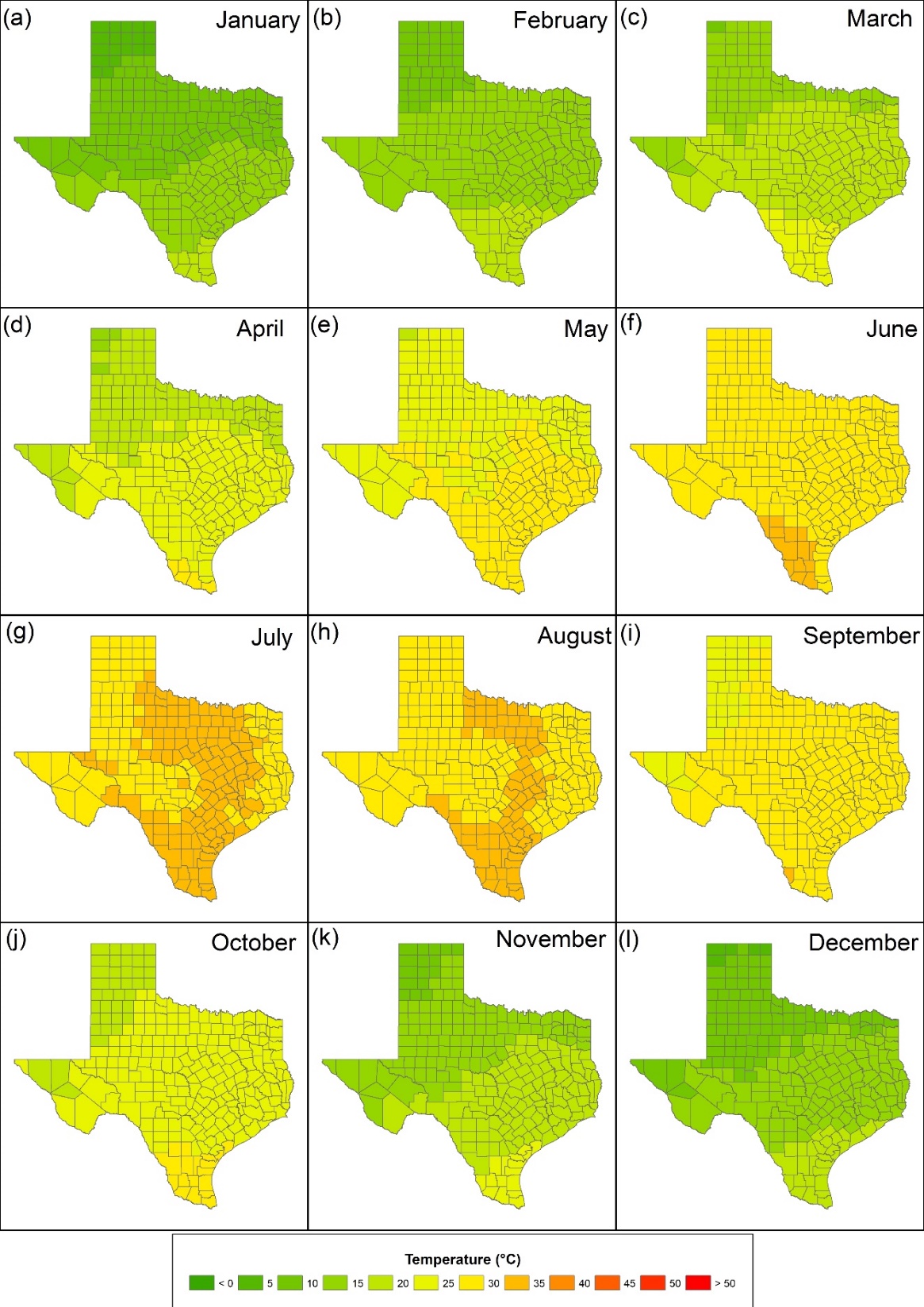


**Figure S1:** Monthly mean temperature projections (°C) for The Canadian Earth System Model version 5 with SSP 1 for 2021 to 2040 aggregated by county.


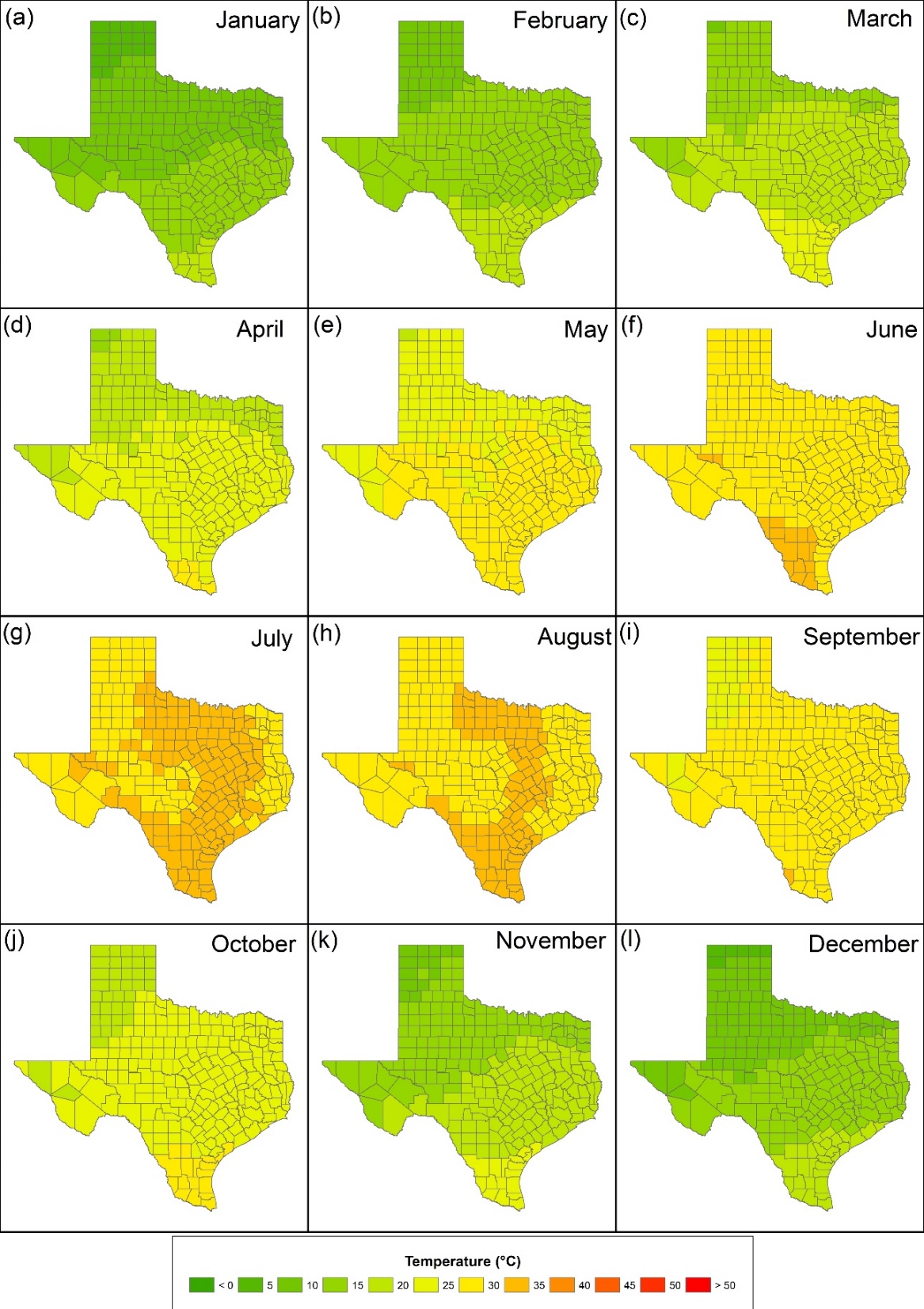


**Figure S2:** Monthly mean temperature projections (°C) for The Canadian Earth System Model version 5 with SSP 2 for 2021 to 2040 aggregated by county.


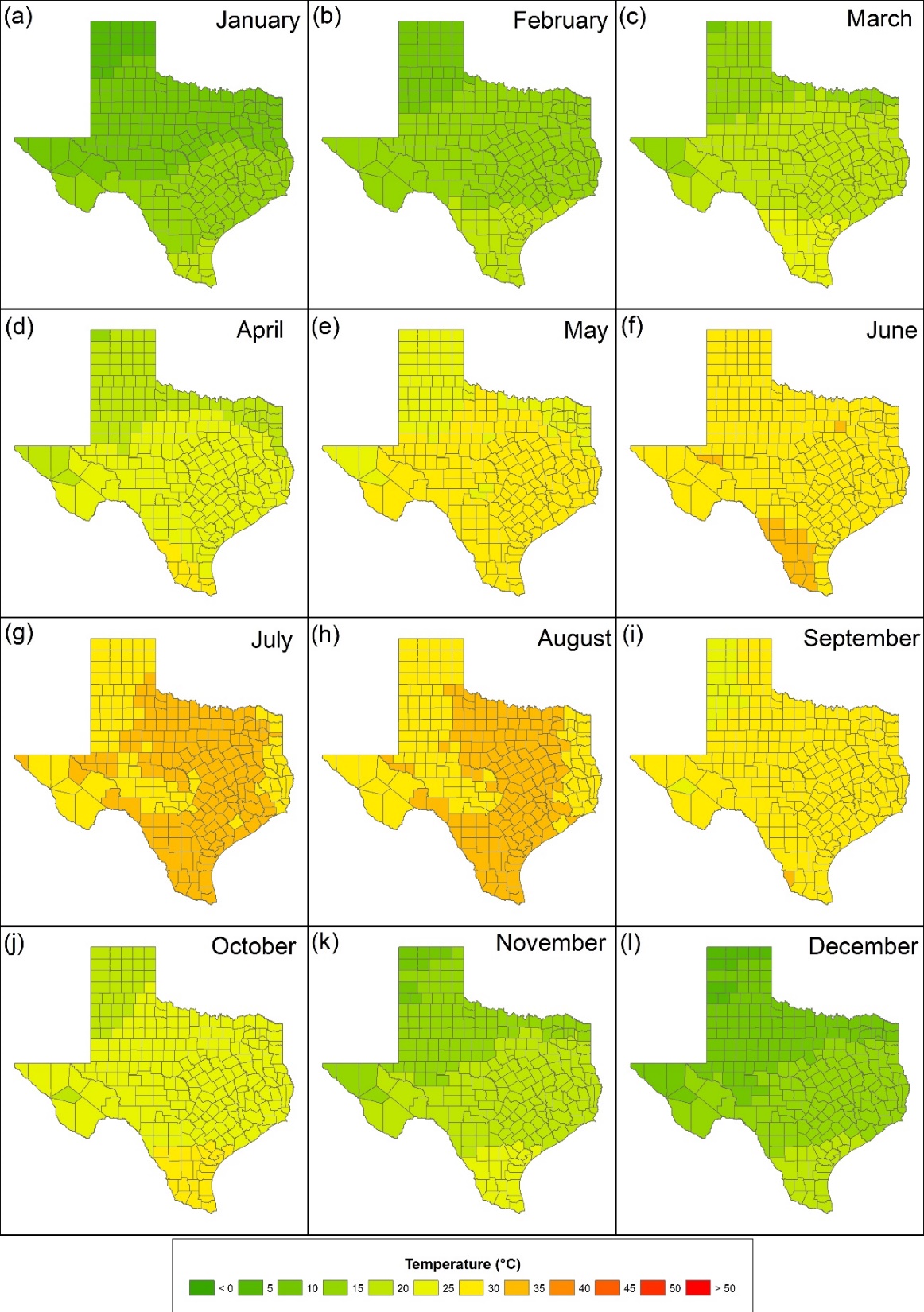


**Figure S3:** Monthly mean temperature projections (°C) for The Canadian Earth System Model version 5 with SSP 3 for 2021 to 2040 aggregated by county.


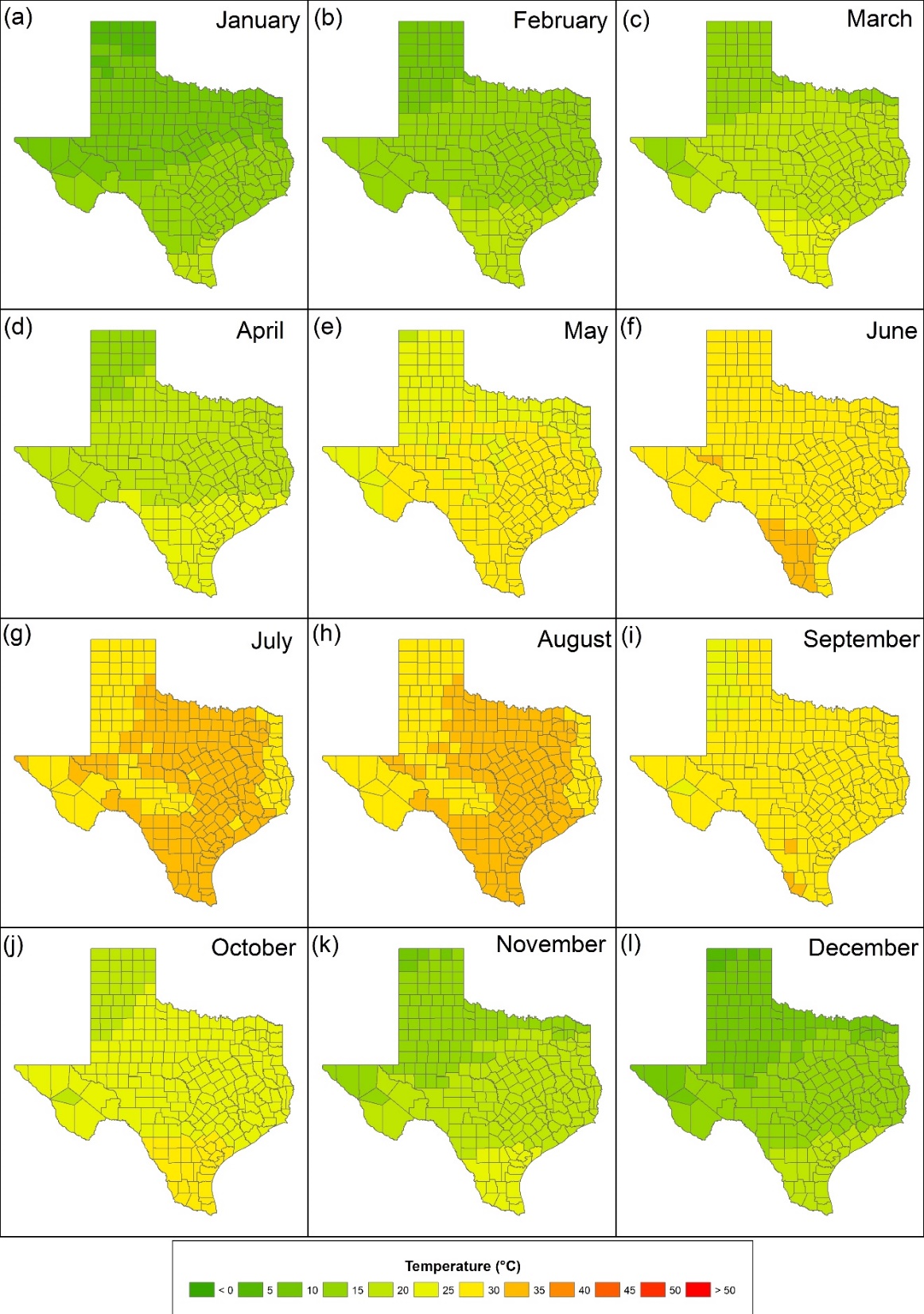


**Figure S4:** Monthly mean temperature projections (°C) for The Canadian Earth System Model version 5 with SSP 5 for 2021 to 2040 aggregated by county.


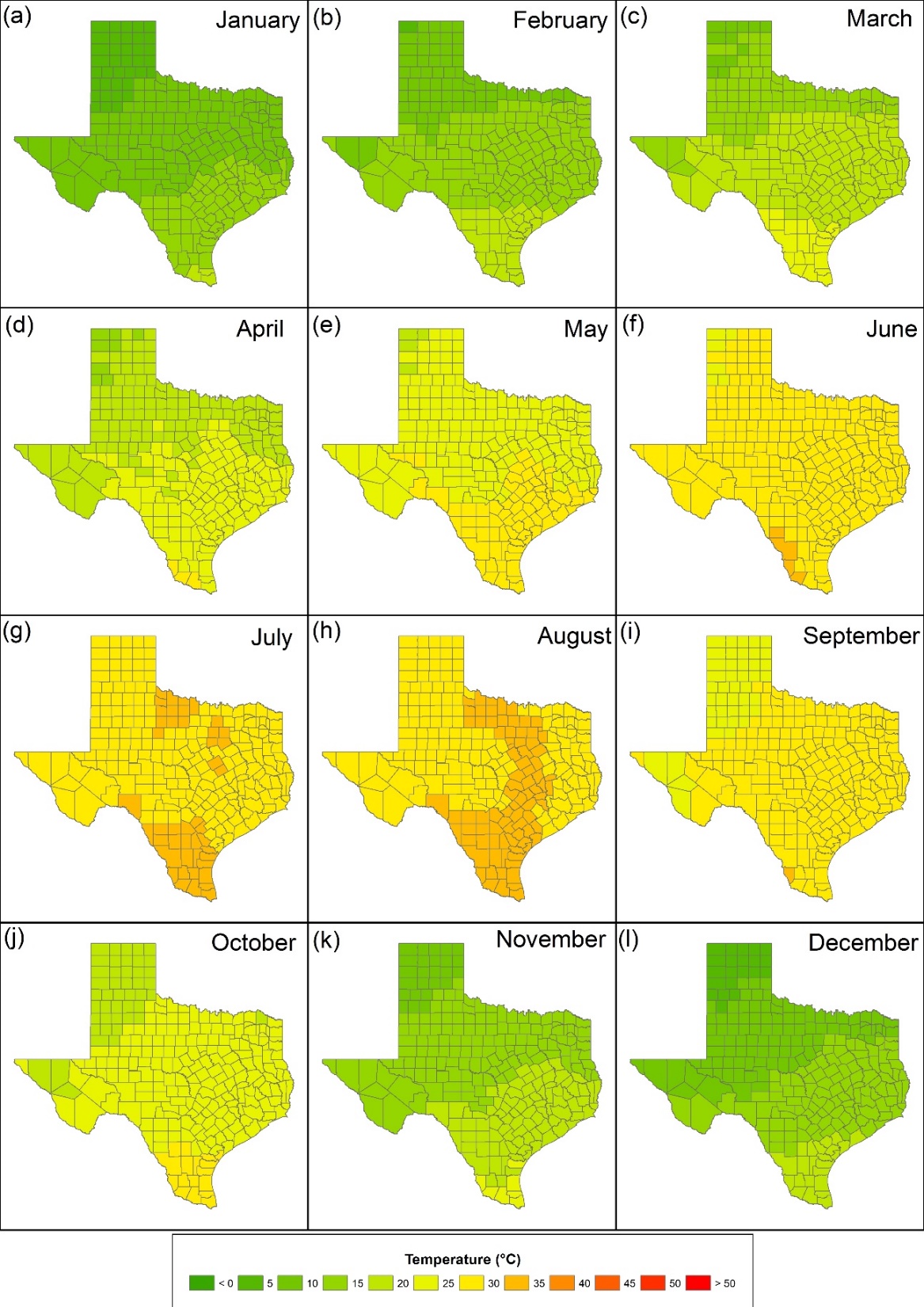


**Figure S5:** Monthly mean temperature projections (°C) for The Institut Pierre-Simon Laplace- Climate Model version 6A- Low Resolution with SSP 1 for 2021 to 2040 aggregated by county.


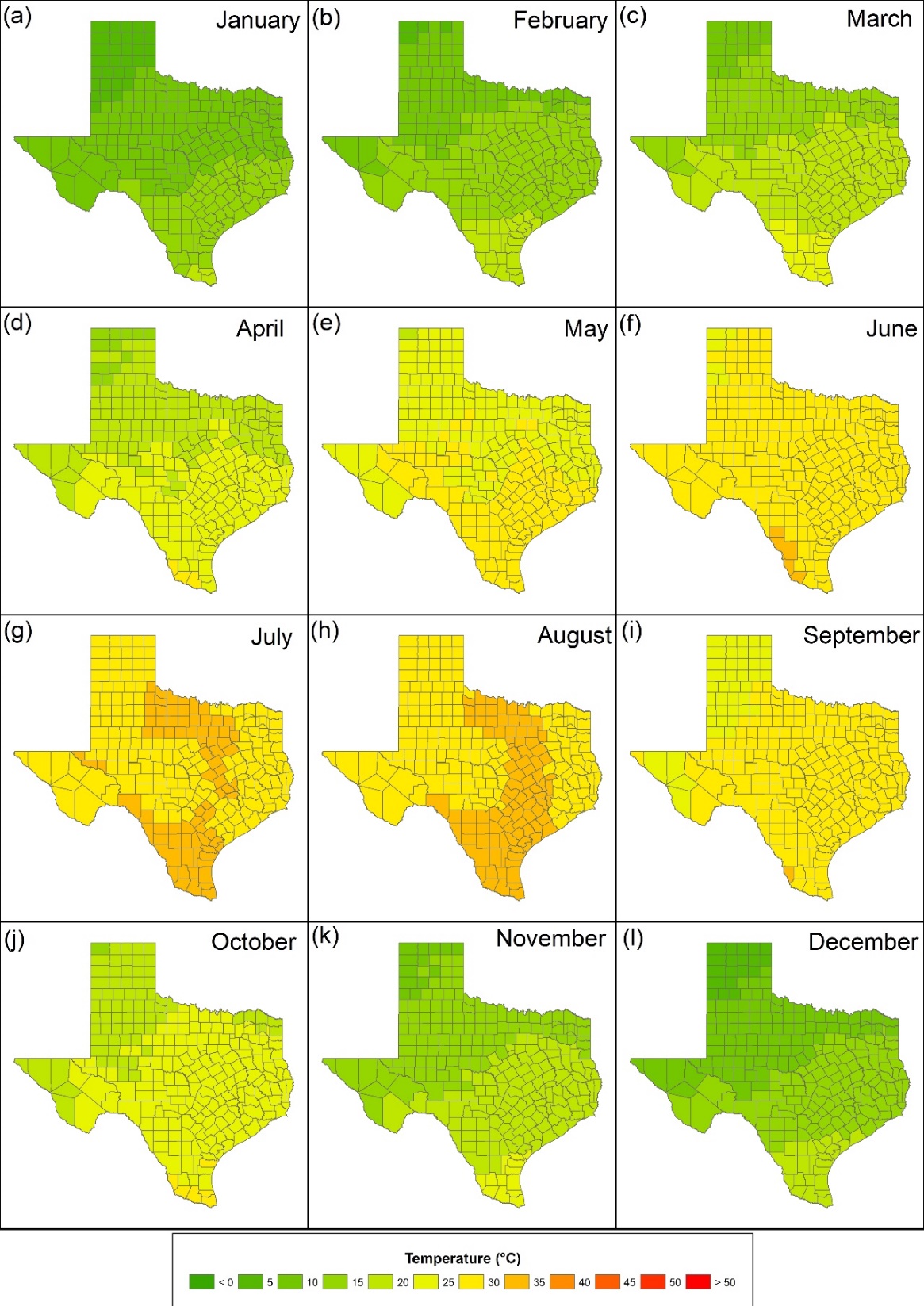


**Figure S6:** Monthly mean temperature projections (°C) for The Institut Pierre-Simon Laplace- Climate Model version 6A- Low Resolution with SSP 2 for 2021 to 2040 aggregated by county.


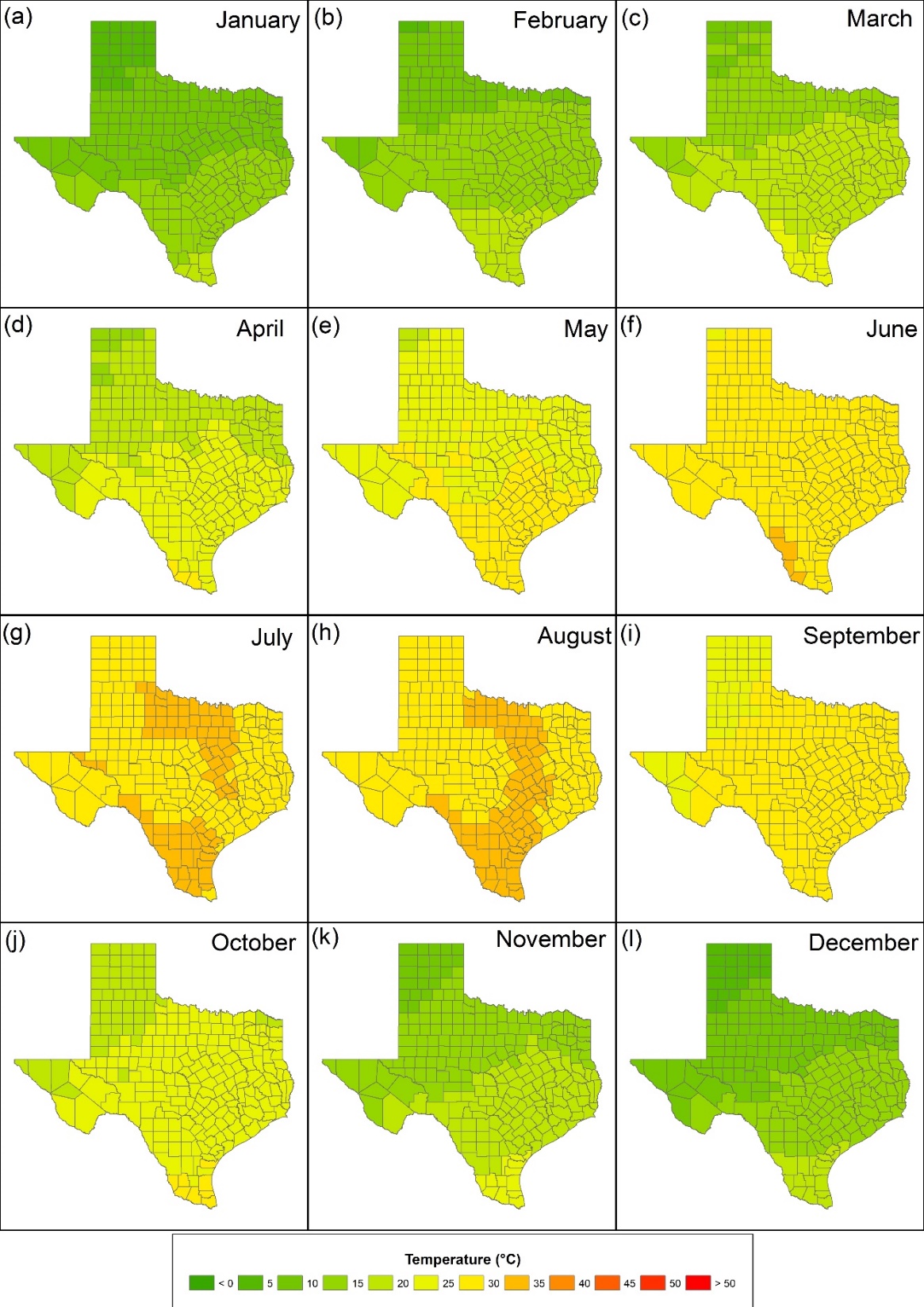


**Figure S7:** Monthly mean temperature projections (°C) for The Institut Pierre-Simon Laplace- Climate Model version 6A- Low Resolution with SSP 3 for 2021 to 2040 aggregated by county.


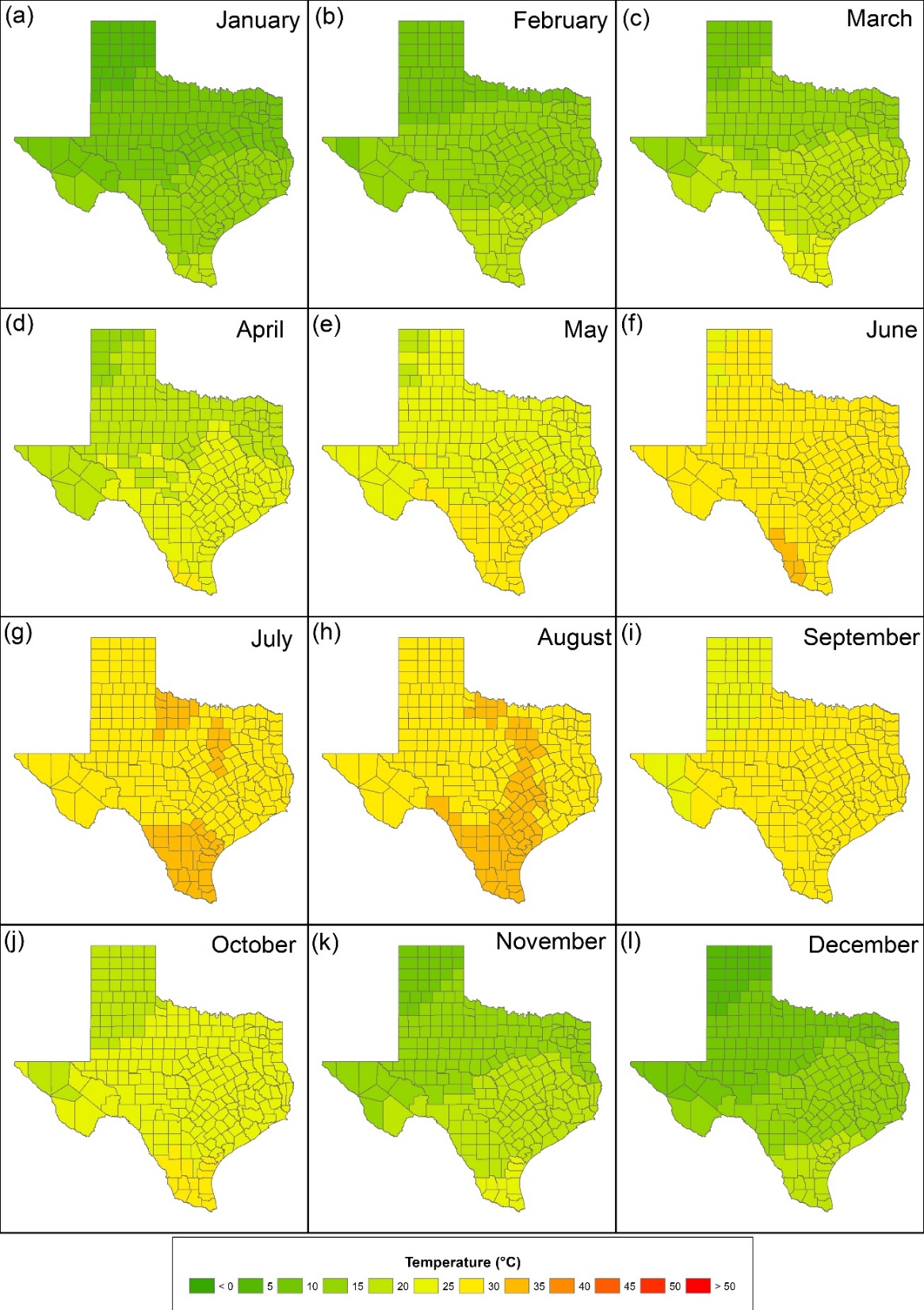


**Figure S8:** Monthly mean temperature projections (°C) for The Institut Pierre-Simon Laplace- Climate Model version 6A- Low Resolution with SSP 5 for 2021 to 2040 aggregated by county.


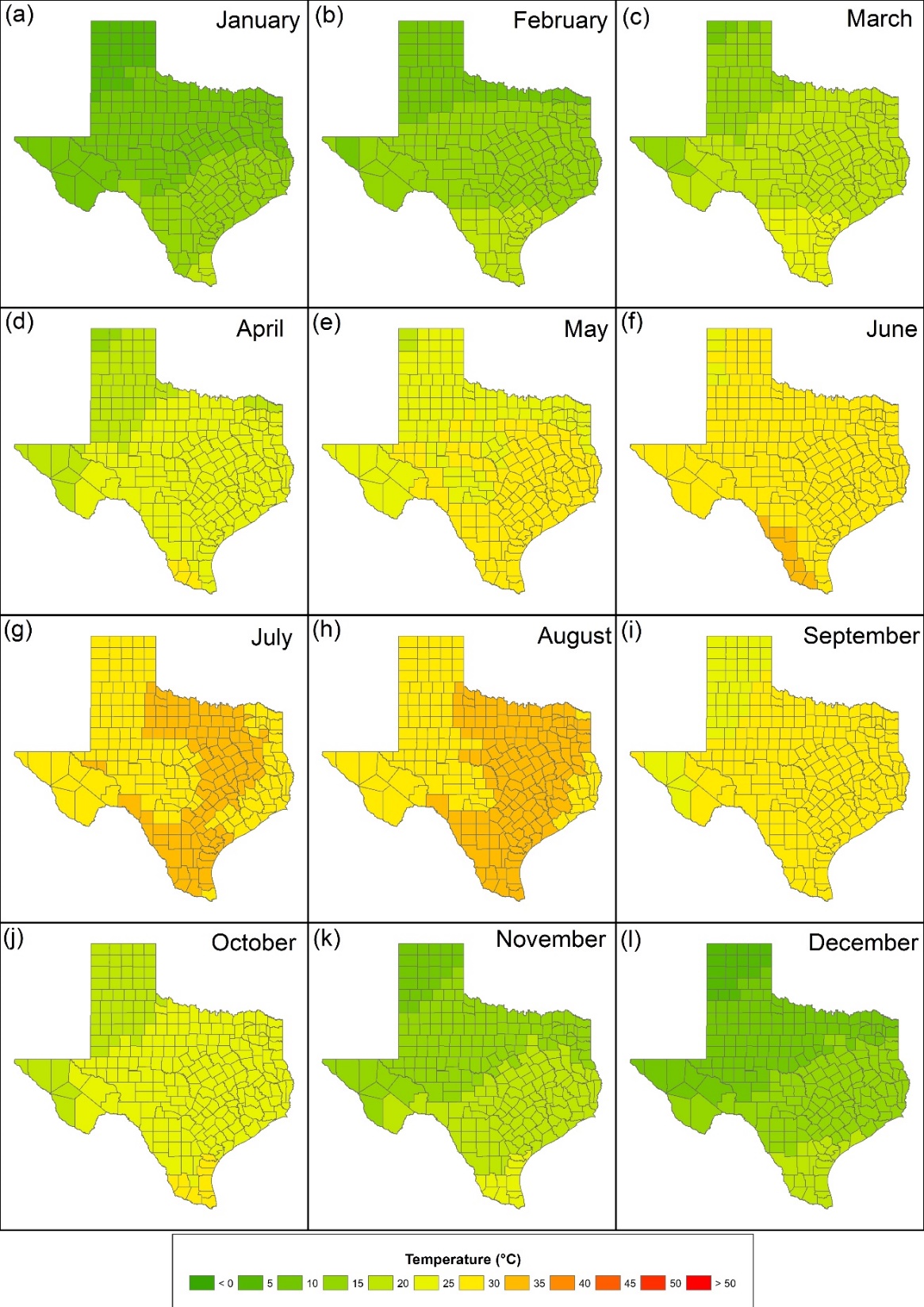


**Figure S9:** Monthly mean temperature projections (°C) for The Model for Interdisciplinary Research on Climate, Earth System version 2 for Long-term simulations with SSP 1 for 2021 to 2040 aggregated by county.


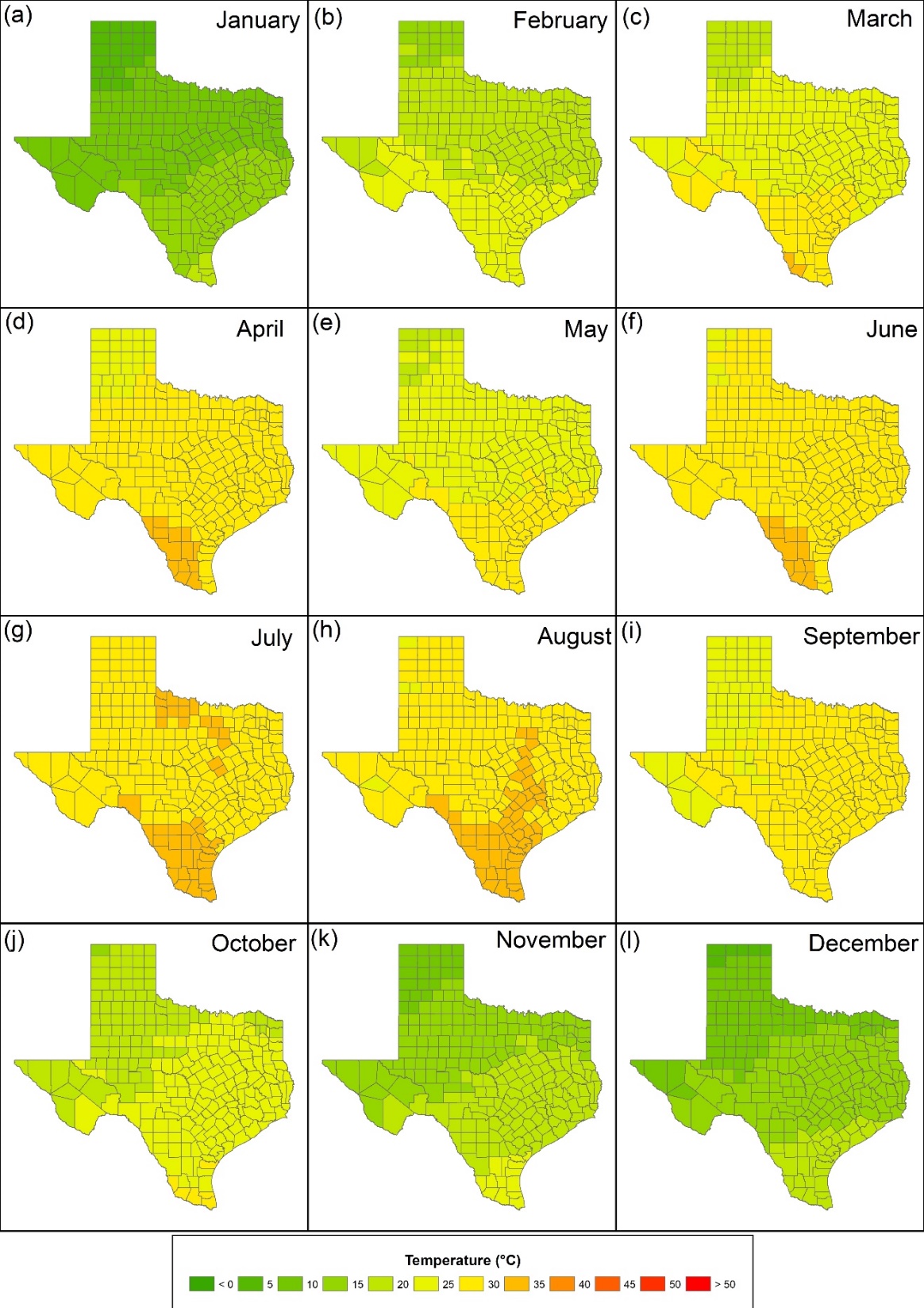


**Figure S10:** Monthly mean temperature projections (°C) for The Model for Interdisciplinary Research on Climate, Earth System version 2 for Long-term simulations with SSP 2 for 2021 to 2040 aggregated by county.


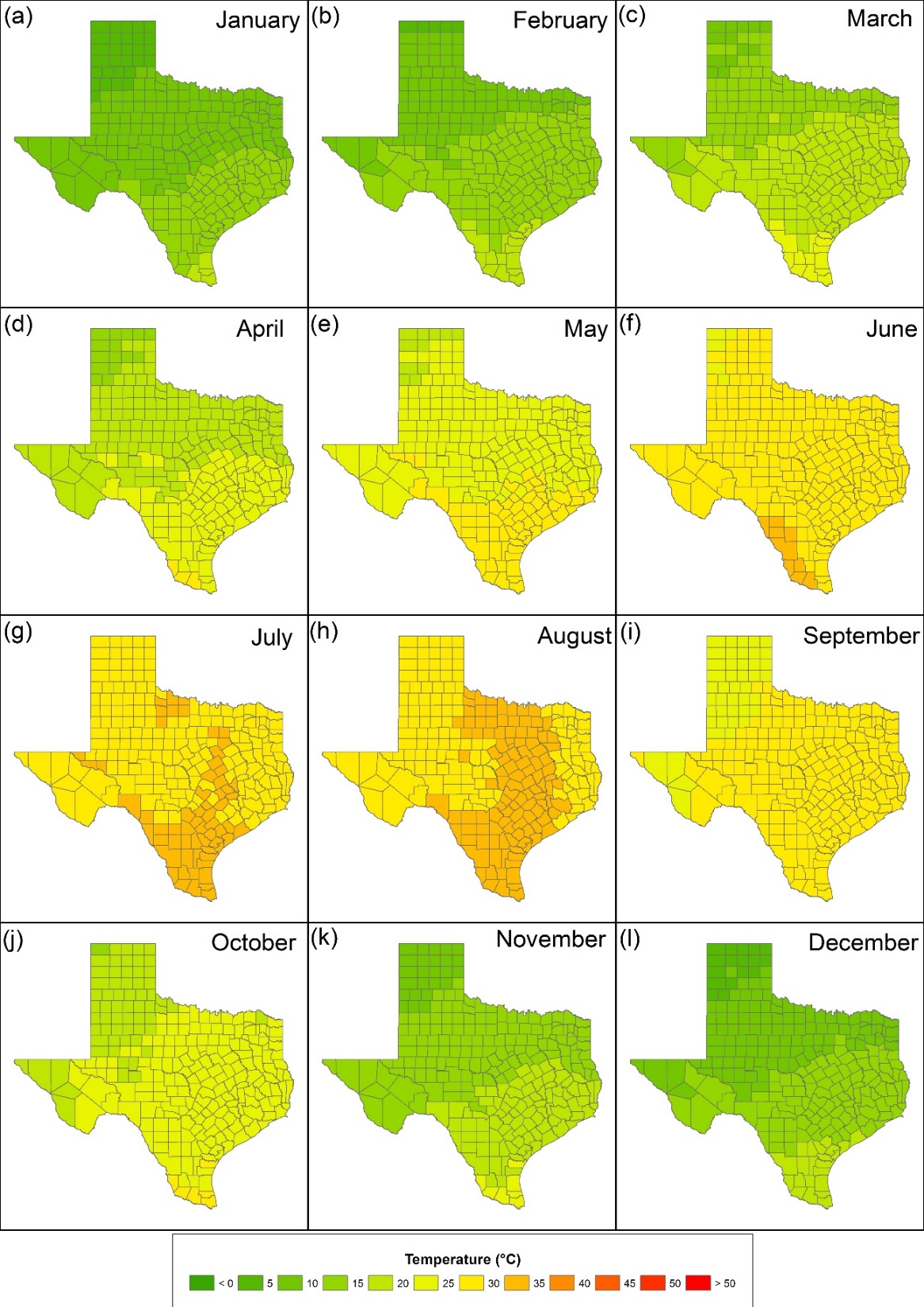


**Figure S11:** Monthly mean temperature projections (°C) for The Model for Interdisciplinary Research on Climate, Earth System version 2 for Long-term simulations with SSP 3 for 2021 to 2040 aggregated by county.


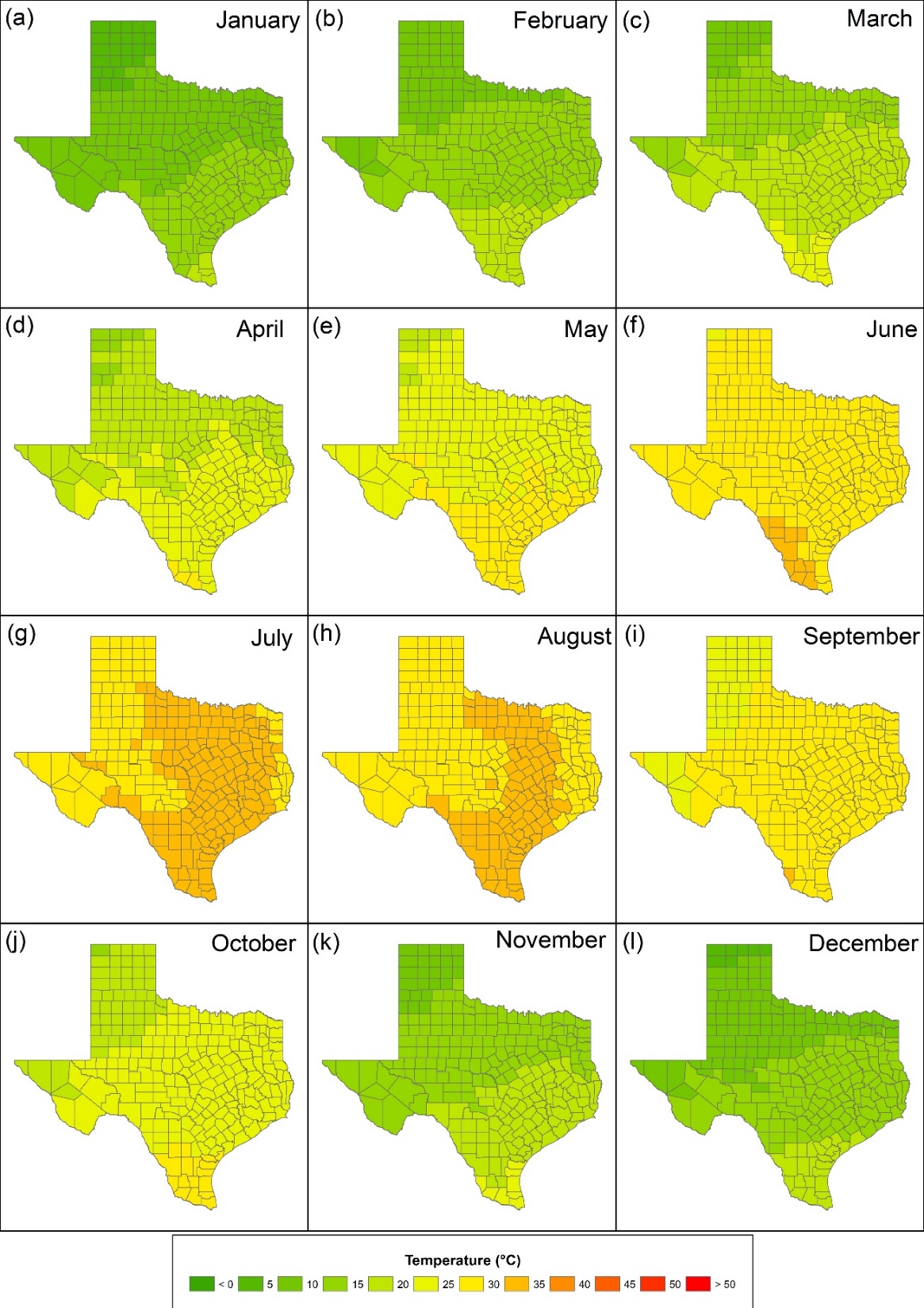


**Figure S12:** Monthly mean temperature projections (°C) for The Model for Interdisciplinary Research on Climate, Earth System version 2 for Long-term simulations with SSP 5 for 2021 to 2040 aggregated by county.


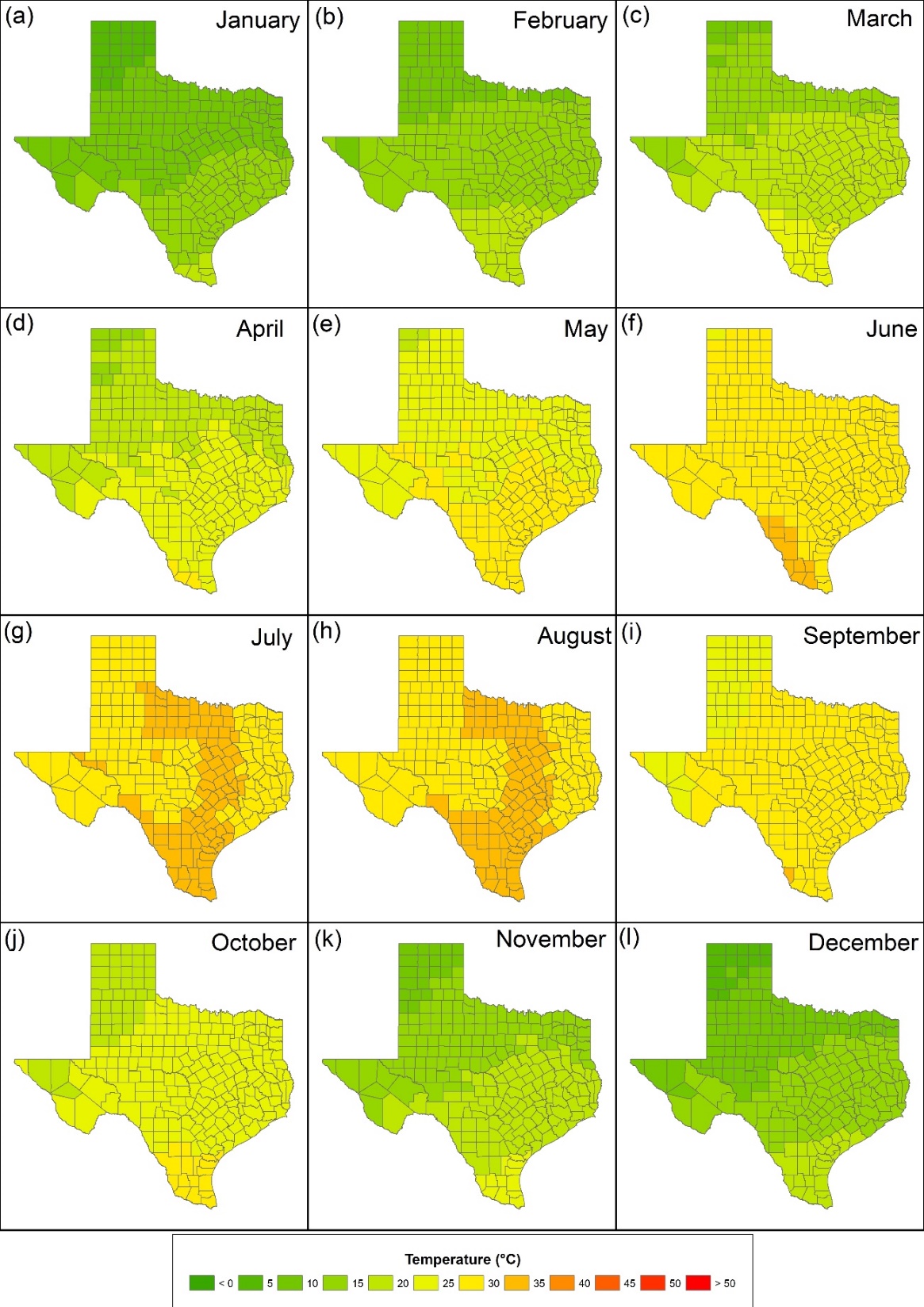


**Figure S13:** Monthly mean temperature projections (°C) for the Ensemble model for 2021 to 2040 aggregated by county.


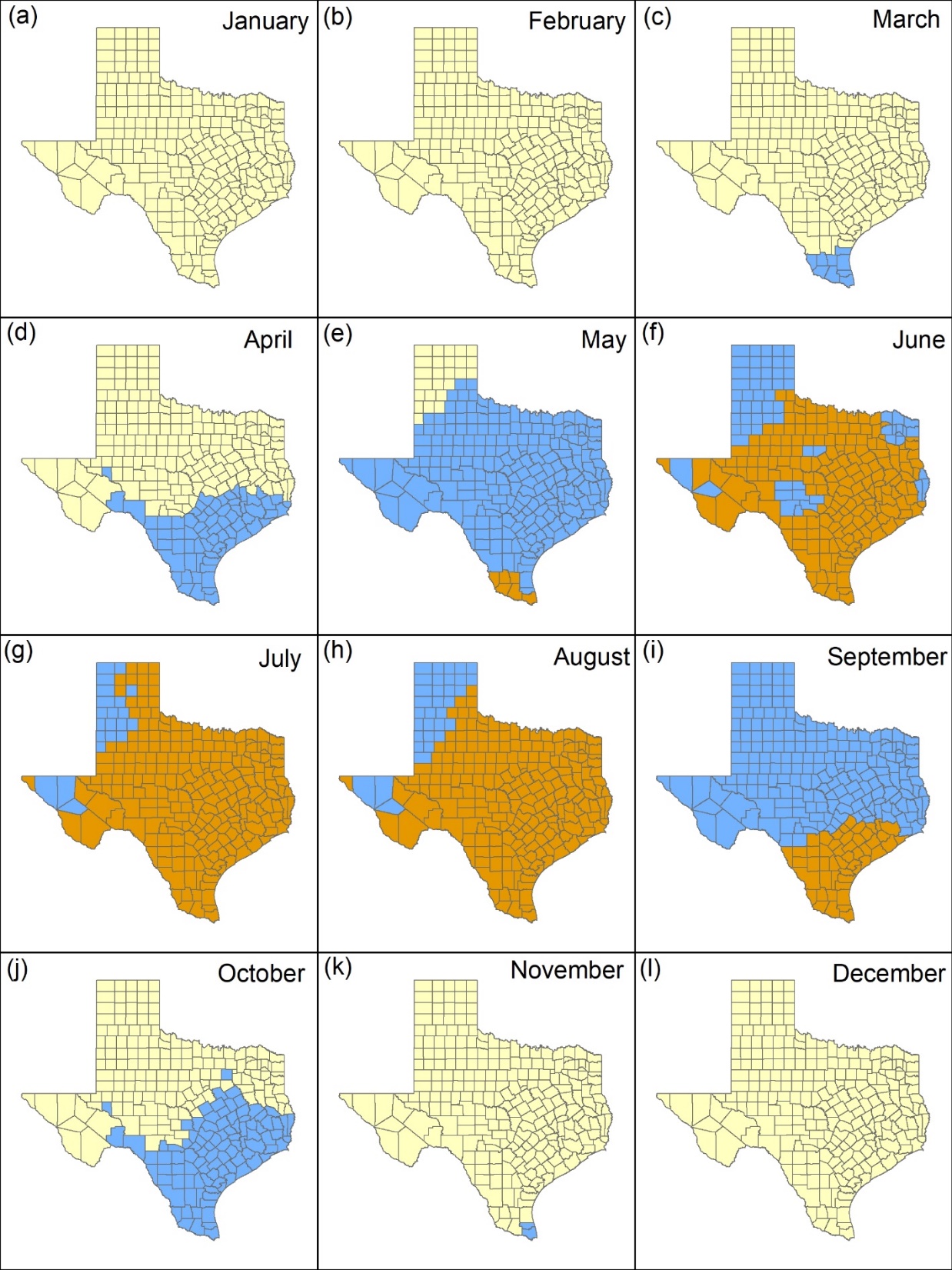


**Figure S14:** Monthly mean temperature (°C) from 2010 to 2019 for Texas aggregated by county. Yellow counties represent areas where temperatures are below the maximum clear ambient temperature in which deer can be detected (≤ 20°C), blue counties represent areas where temperatures are below the maximum cloudy ambient temperature in which deer can be detected (≤ 27°C), and orange counties represent areas where temperatures are above the maximum ambient temperatures for deer detection (> 27°C).


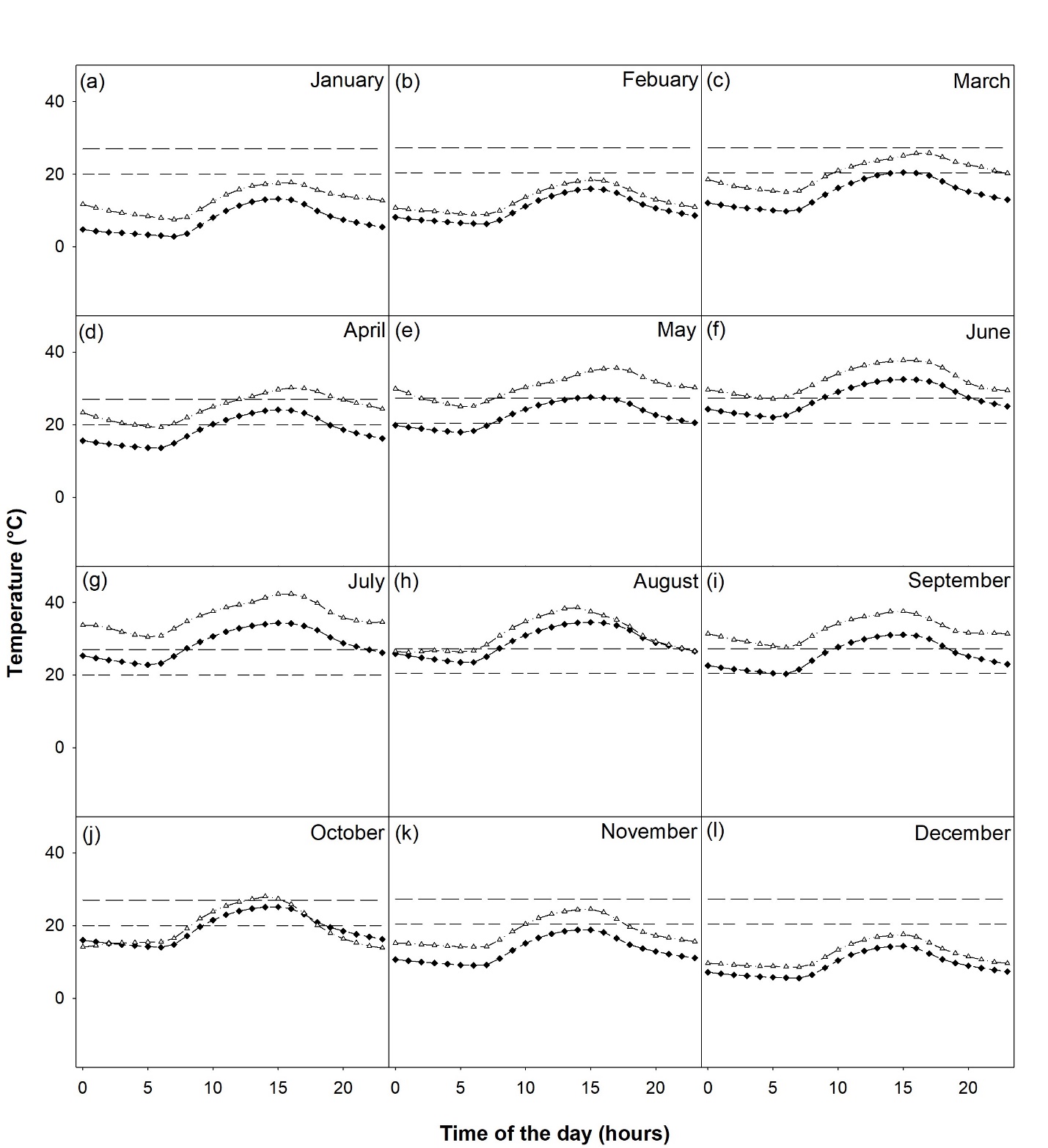
**Figure S15:** Cross Timbers ecoregion hourly mean temperature by month between 2010 and 2019 (black diamonds) and projections to 2080 (white triangles). The double dashed line (20°C) represents maximum clear ambient temperature and the single dashed line (27°C) represents the maximum cloudy ambient temperature for deer detections.


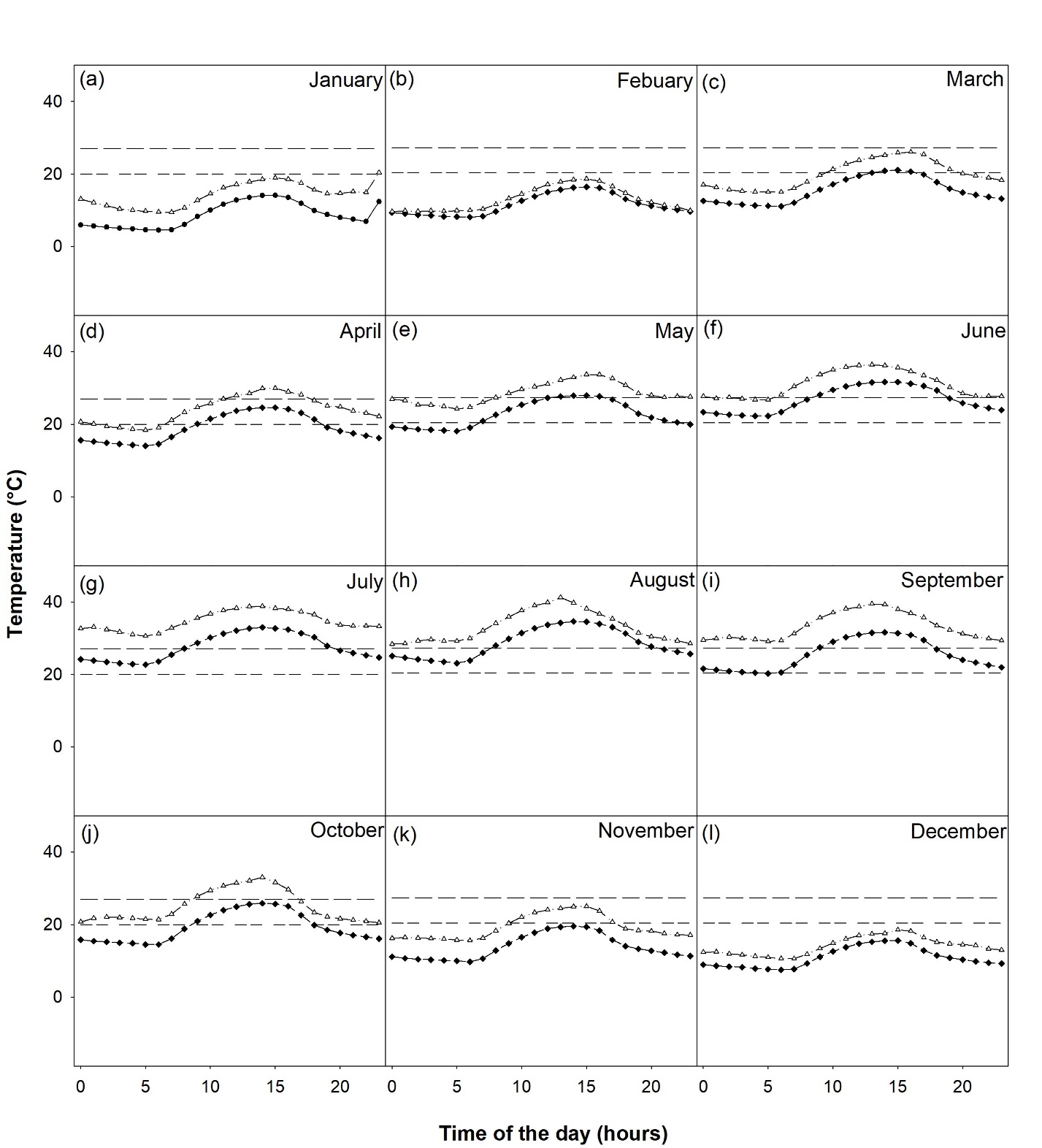
**Figure S16:** Piney Woods ecoregion hourly mean temperature by month between 2010 and 2019 (black diamonds) and projections to 2080 (white triangles). The double dashed line (20°C) represents maximum clear ambient temperature and the single dashed line (27°C) represents the maximum cloudy ambient temperature for deer detections.


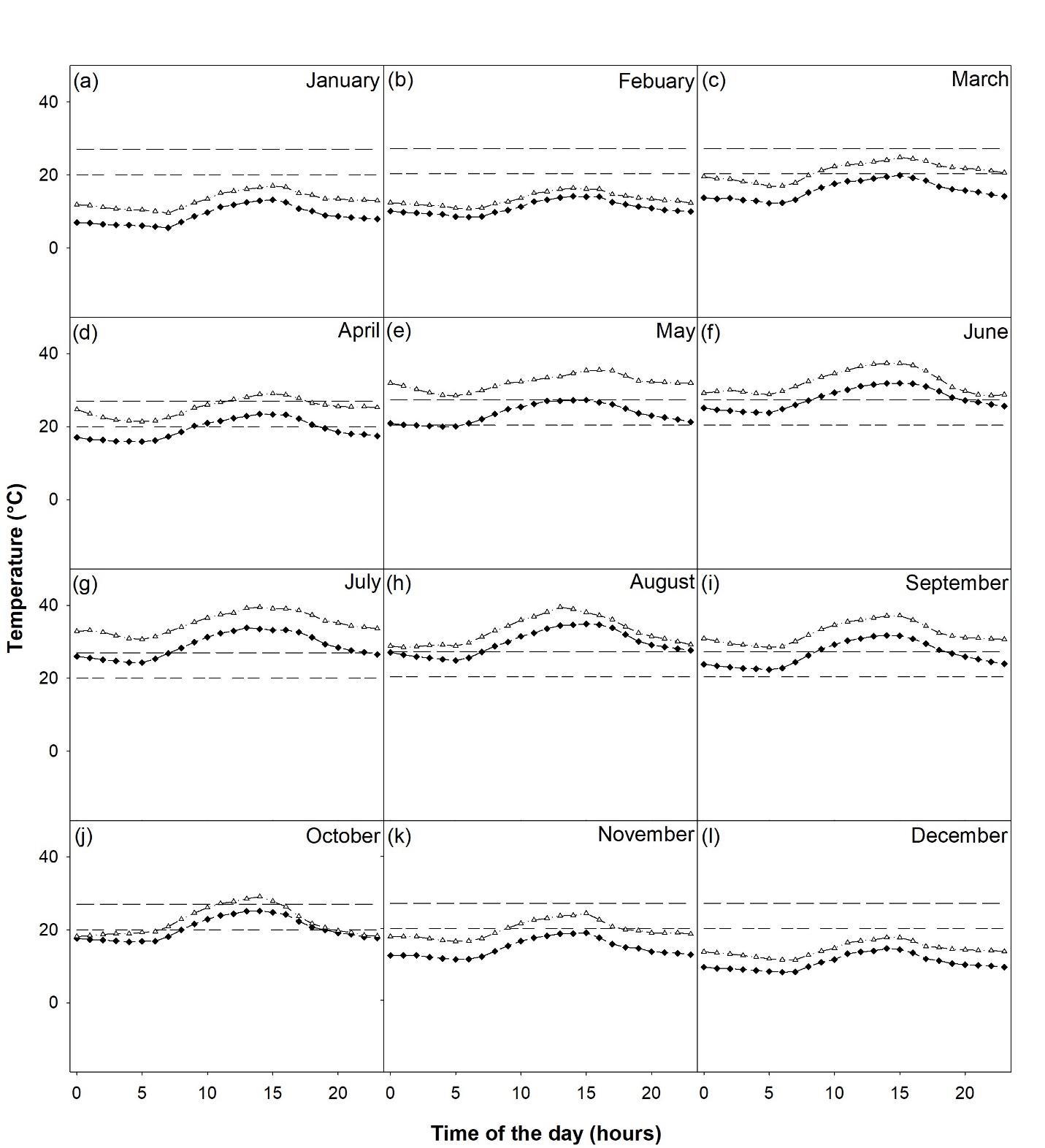
 **Figure S17:** Blackland Prairie ecoregion hourly mean temperature by month between 2010 and 2019 (black diamonds) and projections to 2080 (white triangles). The double dashed line (20°C) represents maximum clear ambient temperature and the single dashed line (27°C) represents the maximum cloudy ambient temperature for deer detections.


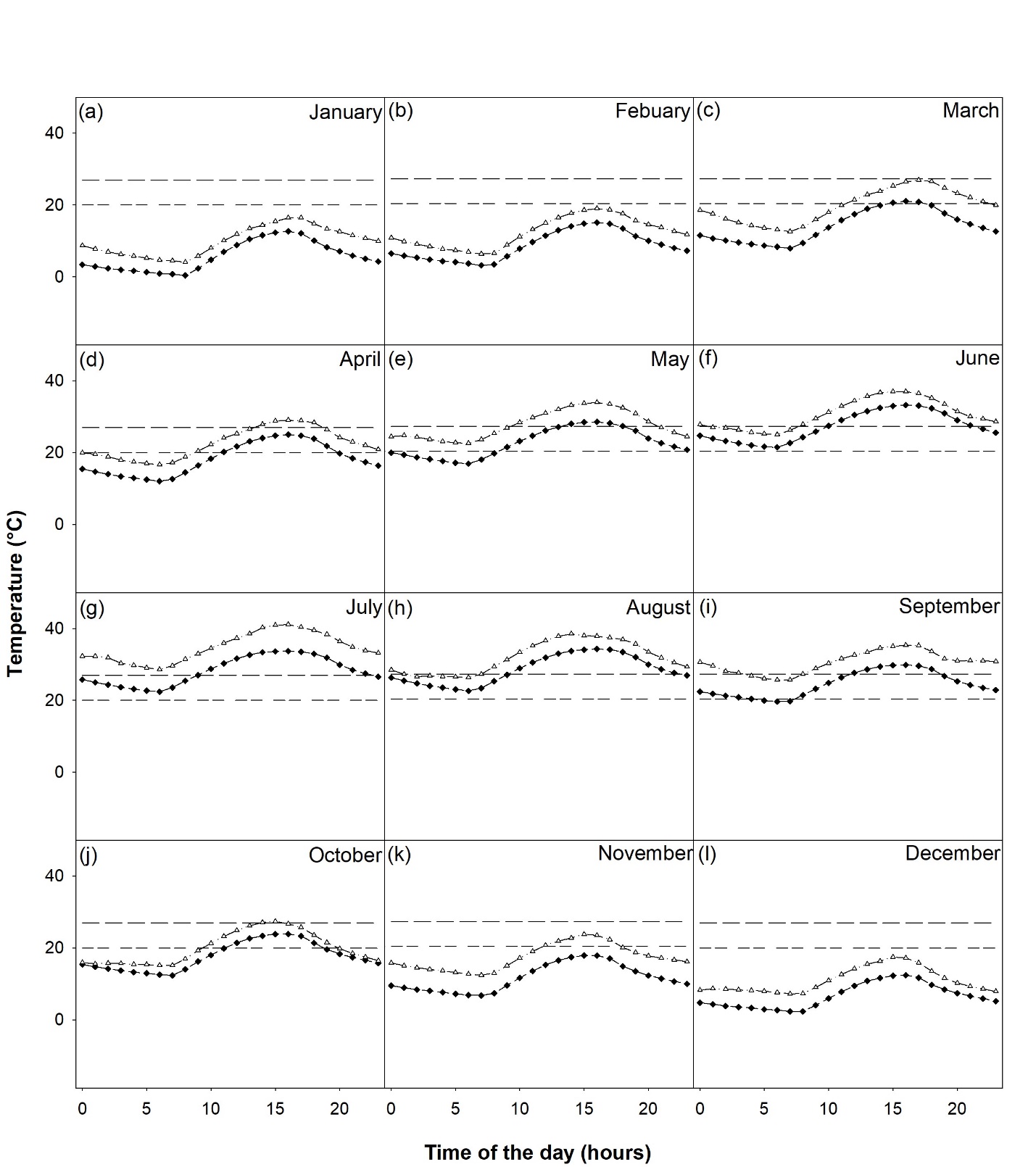
**Figure S18:** Rolling Plains ecoregion hourly mean temperature by month between 2010 and 2019 (black diamonds) and projections to 2080 (white triangles). The double dashed line (20°C) represents maximum clear ambient temperature and the single dashed line (27°C) represents the maximum cloudy ambient temperature for deer detections.


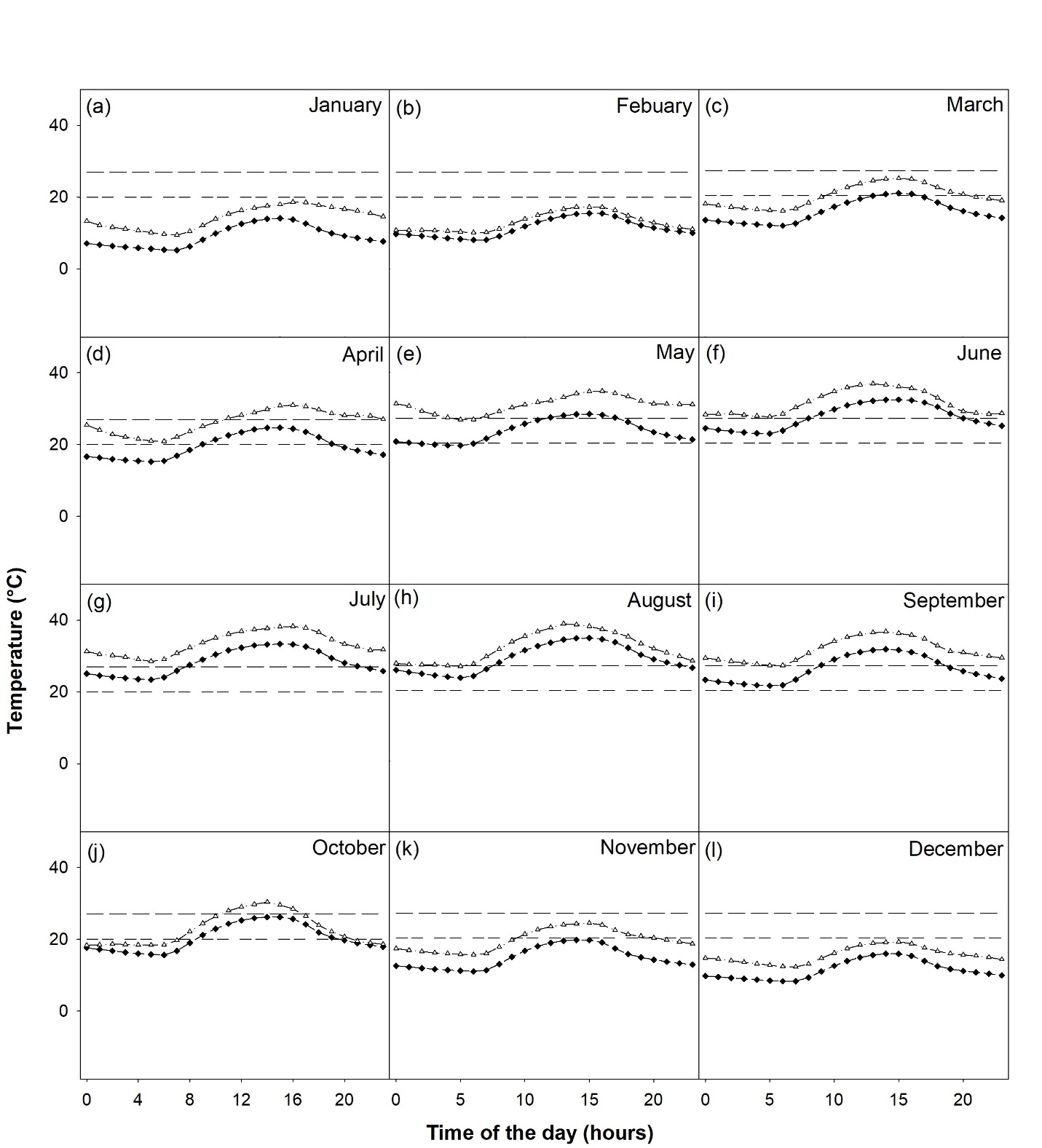
**Figure S19:** Post Oak Savannah ecoregion hourly mean temperature by month between 2010 and 2019 (black diamonds) and projections to 2080 (white triangles). The double dashed line (20°C) represents maximum clear ambient temperature and the single dashed line (27°C) represents the maximum cloudy ambient temperature for deer detections.


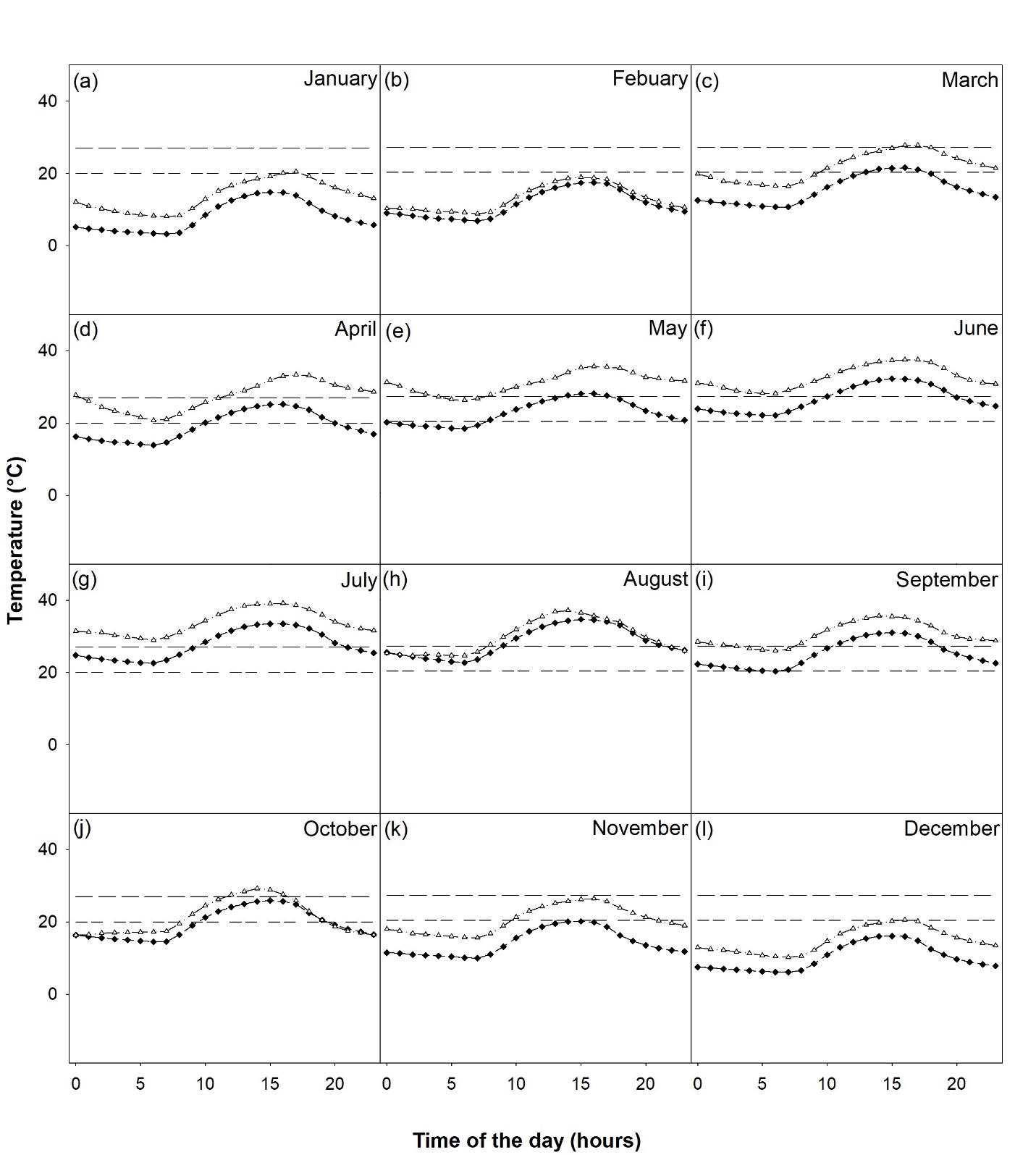
**Figure S20:** Edwards Plateau ecoregion hourly mean temperature by month between 2010 and 2019 (black diamonds) and projections to 2080 (white triangles). The double dashed line (20°C) represents maximum clear ambient temperature and the single dashed line (27°C) represents the maximum cloudy ambient temperature for deer detections.


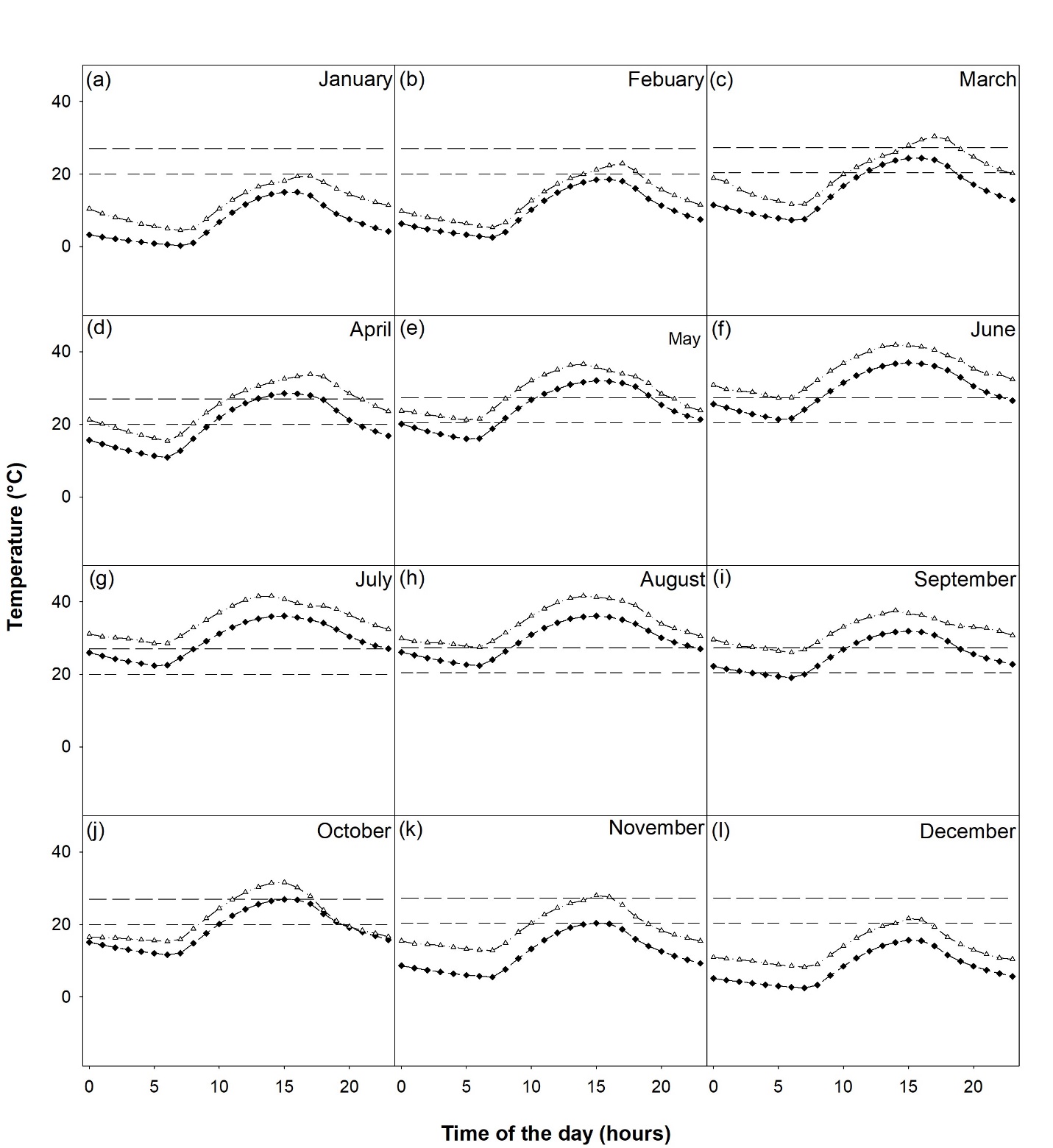
**Figure S21:** Trans-Pecos ecoregion hourly mean temperature by month between 2010 and 2019 (black diamonds) and projections to 2080 (white triangles). The double dashed line (20°C) represents maximum clear ambient temperature and the single dashed line (27°C) represents the maximum cloudy ambient temperature for deer detections.


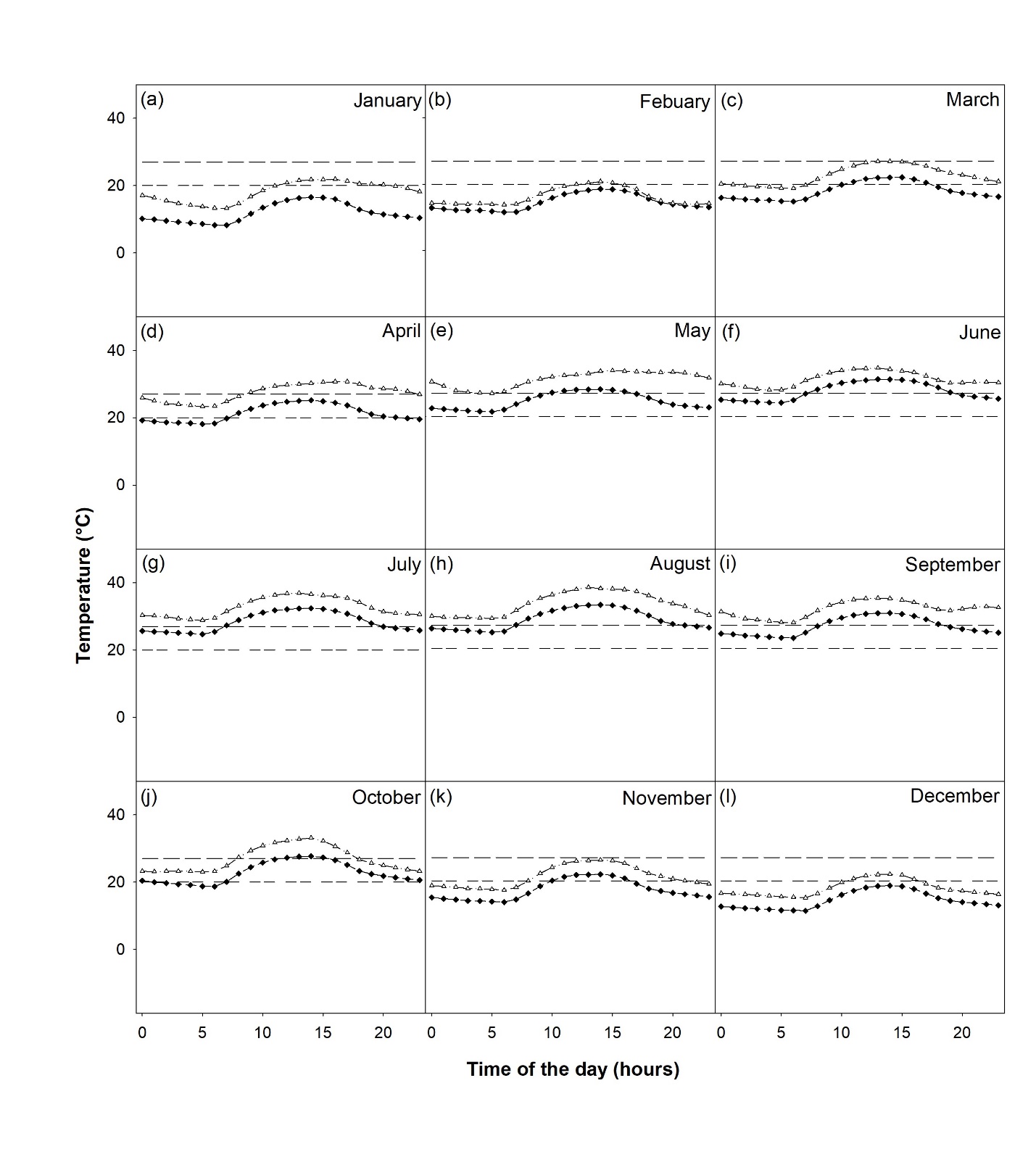
**Figure S22:** Gulf Prairies ecoregion hourly mean temperature by month between 2010 and 2019 (black diamonds) and projections to 2080 (white triangles). The double dashed line (20°C) represents maximum clear ambient temperature and the single dashed line (27°C) represents the maximum cloudy ambient temperature for deer detections.


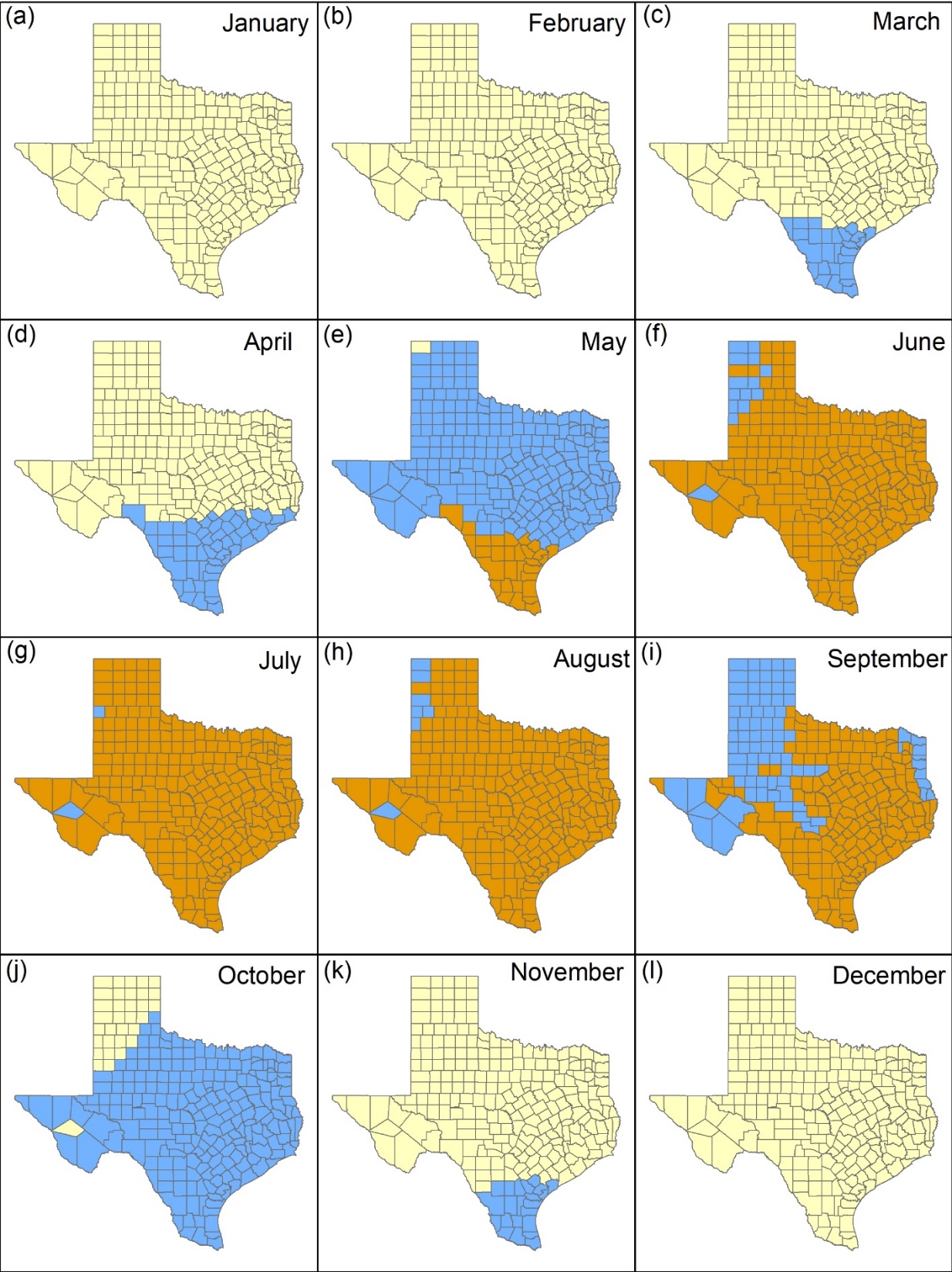


**Figure S23:** Can-ESM5 SSP 5-8.5 model 2021 to 2040 projections aggregated by county. Yellow counties represent areas where temperatures are below the maximum clear ambient temperature in which deer can be detected (≤ 20°C), blue counties represent areas where temperatures are below the maximum cloudy ambient temperature in which deer can be detected (≤ 27°C), and orange counties represent areas where temperatures are above the maximum ambient temperatures for deer detection (> 27°C).


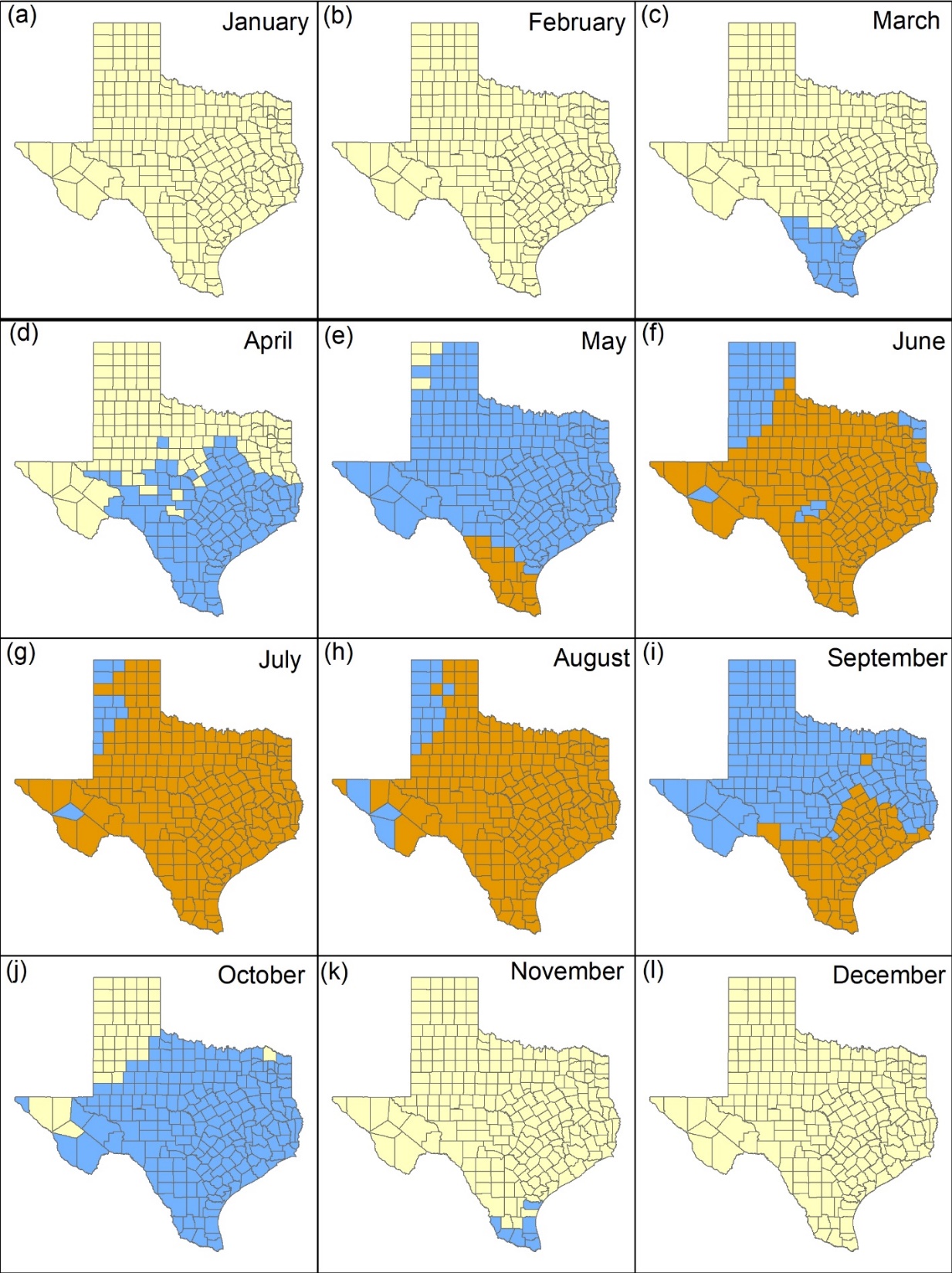


**Figure S24:** IPSL-CM6A-LR SSP 1-2.6 model 2021 to 2040 projections aggregated by county. Yellow counties represent areas where temperatures are below the maximum clear ambient temperature in which deer can be detected (≤ 20°C), blue counties represent areas where temperatures are below the maximum cloudy ambient temperature in which deer can be detected (≤ 27°C), and orange counties represent areas where temperatures are above the maximum ambient temperatures for deer detection (> 27°C).


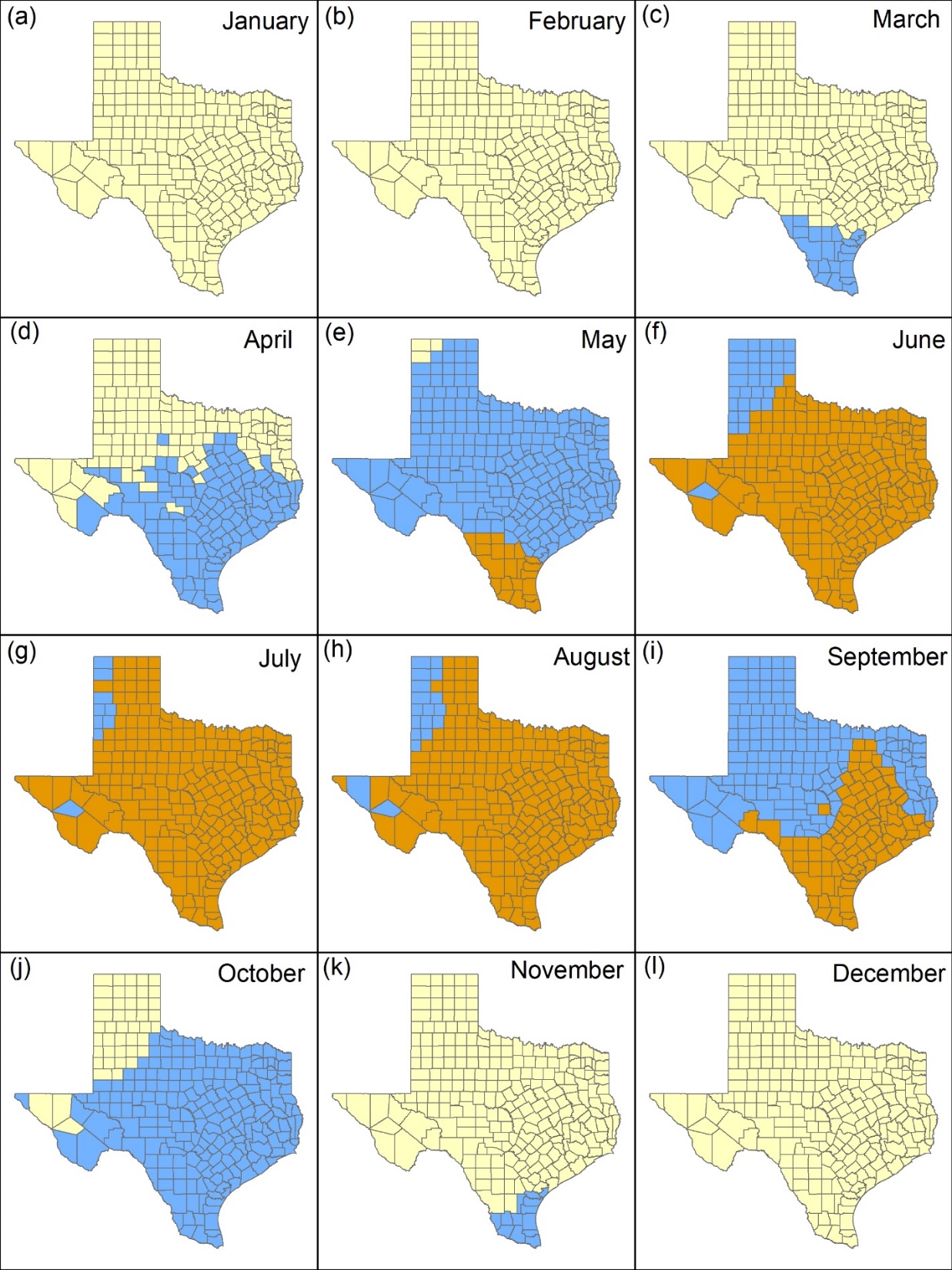


**Figure S25:** Ensemble model 2021 to 2040 projections aggregated by county. Yellow counties represent areas where temperatures are below the maximum clear ambient temperature in which deer can be detected (≤ 20°C), blue counties represent areas where temperatures are below the maximum cloudy ambient temperature in which deer can be detected (≤ 27°C), and orange counties represent areas where temperatures are above the maximum ambient temperatures for deer detection (> 27°C).
